# Disease spectrum and prognostic factors in patients treated for tuberculous meningitis in Shaanxi province, China

**DOI:** 10.3389/fmicb.2024.1374458

**Published:** 2024-05-17

**Authors:** Ting Wang, Meng-yan Li, Xin-shan Cai, Qiu-sheng Cheng, Ze Li, Ting-ting Liu, Lin-fu Zhou, Hong-hao Wang, Guo-dong Feng, Ben J. Marais, Gang Zhao

**Affiliations:** ^1^Department of Neurology, Guangzhou First People's Hospital, School of Medicine, South China University of Technology, Guangzhou, China; ^2^Department of Clinical Laboratory, Guangzhou Chest Hospital, Guangzhou, China; ^3^Department of Neurology, Xijing Hospital, The Air Force Medical University, Xi’an, China; ^4^Department of Neurology, Northwestern University School of Medicine, Xi’an, China; ^5^Department of Neurology, Zhongshan Hospital, Fudan University, Shanghai, China; ^6^Sydney Infectious Diseases Institute (Sydney ID) and the WHO Collaborating Centre in Tuberculosis, University of Sydney, Sydney, NSW, Australia

**Keywords:** tuberculous meningitis, prognostic factors, disease spectrum, diagnostic, CSF

## Abstract

**Background:**

Tuberculous meningitis (TBM) is the most severe form of tuberculosis (TB) and can be difficult to diagnose and treat. We aimed to describe the clinical presentation, diagnosis, disease spectrum, outcome, and prognostic factors of patients treated for TBM in China.

**Methods:**

A multicenter retrospective study was conducted from 2009 to 2019 enrolling all presumptive TBM patients referred to Xijing tertiary Hospital from 27 referral centers in and around Shaanxi province, China. Patients with clinical features suggestive of TBM (abnormal CSF parameters) were included in the study if they had adequate baseline information to be classified as “confirmed,” “probable,” or “possible” TBM according to international consensus TBM criteria and remained in follow-up. Patients with a confirmed alternative diagnosis or severe immune compromise were excluded. Clinical presentation, central nervous system imaging, cerebrospinal fluid (CSF) results, TBM score, and outcome—assessed using the modified Barthel disability index—were recorded and compared.

**Findings:**

A total of 341 presumptive TBM patients met selection criteria; 63 confirmed TBM (25 culture positive, 42 Xpert-MTB/RIF positive), 66 probable TBM, 163 possible TBM, and 49 “not TBM.” Death was associated with BMRC grade III (OR = 5.172; 95%CI: 2.298–11.641), TBM score ≥ 15 (OR = 3.843; 95%CI: 1.372–10.761), age > 60 years (OR = 3.566; 95%CI: 1.022–12.442), and CSF neutrophil ratio ≥ 25% (OR = 2.298; 95%CI: 1.027–5.139). Among those with confirmed TBM, nearly one-third (17/63, 27.0%) had a TBM score < 12; these patients exhibited less classic meningitis symptoms and signs and had better outcomes compared with those with a TBM score ≥ 12. In this group, signs of disseminated/miliary TB (OR = 12.427; 95%CI: 1.138–135.758) and a higher TBM score (≥15, OR = 8.437; 95%CI: 1.328–53.585) were most strongly associated with death.

**Conclusion:**

TBM patients who are older (>60 years) have higher TBM scores or CSF neutrophil ratios, have signs of disseminated/miliary TB, and are at greatest risk of death. In general, more effort needs to be done to improve early diagnosis and treatment outcome in TBM patients.

## Introduction

Tuberculous meningitis (TBM) is the most severe form of tuberculosis (TB). The best way to improve TBM outcome is early diagnosis and timely effective treatment (Donovan et al., 2020). Most patients with TBM are diagnosed based on their clinical features, neuroimaging findings, and characteristic changes in their cerebrospinal fluid (CSF). The methods that are currently available for diagnosis (Wang et al., 2016) have their strengths and weaknesses (Marais et al., 2010) but remain sub-optimal (Ho et al., 2013; Seddon and Thwaites, 2019). Difficulties include frequent atypical clinical manifestations, the need for invasive CSF sampling, and poor microbiological yield (Arshad et al., 2020). Therefore, early diagnosis and timely treatment of TBM remains challenging (Huynh et al., 2022).

Microbiological confirmation requires *M. tuberculosis* to be cultured from CSF or a positive World Health Organization (WHO)-approved commercial nucleic acid amplification test (NAAT) (Marais et al., 2010). CSF culture is limited by low sensitivity and slow turn-around time. Liquid culture using the mycobacteria growth indicator tube (MGIT) method is more sensitive and faster than traditional Lowenstein–Jensen (LJ) solid medium used in most TB endemic settings (Koh et al., 2012; Tayyab et al., 2018). Commercial NAATs, such as Xpert MTB/RIF and Xpert MTB/RIF Ultra (Dorman et al., 2018; Kohli et al., 2021), have sensitivity comparable to culture and can be completed within 2 h, but it is relatively expensive. Although it is extremely useful as a ‘rule in’ test, its sensitivity is too low to serve as a reliable ‘rule out’ test (Kohli et al., 2021). Microscopic acid-fast staining, including modified Ziehl–Neelsen (MZN) staining (Chen et al., 2012), is convenient and fast but hampered by poor accuracy (Wang et al., 2016).

To improve TBM management in China, it is important for doctors to consider the relative value of different diagnostic approaches and the factors associated with poor outcome. Therefore, we conducted a study to describe the clinical presentation and outcome of patients treated for TBM, with specific emphasis on diagnostic approaches and prognostic factors.

## Materials and methods

### Study design, setting, and ethics approval

We performed a multicenter retrospective study from May 2009 to April 2019, including all patients with presumptive TBM referred to Xijing tertiary Hospital from 27 referral centers in and around Shaanxi province, China, which was coordinated by Xijing hospital of the Air Force Medical University; one of the largest medical centers in China (a 3,218-bed university-affiliated hospital). All patients underwent chest X-ray (CXR), lumber puncture, brain magnetic resonance imaging (MRI), and/or computed tomography (CT) scanning and were tested for human immunodeficiency virus (HIV) using an enzyme-linked immunosorbent assay. The study protocol was approved by the Ethics Committee of Xijing Hospital of Air Force Medical University (Study No. KY20105255-1 and No. KY20163367-1) and the Ethics Committee of Guangzhou First People’s Hospital (Study No. K-2022-054-01).

### Clinical data

**Figure 1:**
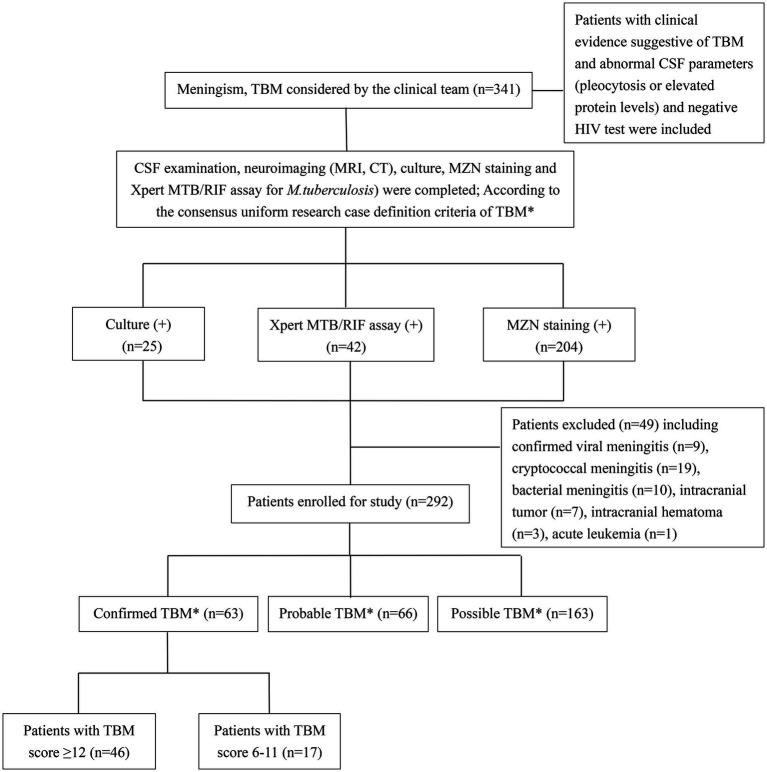
Flow diagram of patients with presumptive tuberculous meningitis included in the study. TBM, tuberculous meningitis; CSF, cerebrospinal fluid; MZN, modified Ziehl–Neelsen staining; HIV, human immunodeficiency virus. *According to consensus uniform research case definition criteria (Marais et al., 2010) (Supplementary Panel S1).

Clinical data (including demographics, clinical, radiological, and routine CSF laboratory results) were collected through medical record review using a standard data capture tool, while trained interviewers completed telephone follow-ups after the patient was discharged from hospital on TB treatment using a standard questionnaire. Patients with clinical signs and symptoms and abnormal CSF parameters (pleocytosis or elevated protein levels) suggestive of TBM are classified as “confirmed,” “probable,” or “possible” TBM based on international uniform consensus diagnostic criteria for TBM research (Marais et al., 2010) (Supplementary Panel S1). Patients were only included if meet minimum data quality and completeness criteria were met. Confirmed TBM required a positive *M. tuberculosis* culture or Xpert MTB/RIF on CSF, and given that all patients received brain imaging, probable TBM required a TBM score of ≥12 and possible TBM score of 6–11. Severity grading was done using revised British Medical Research Council (BMRC, 1948) TBM severity grade criteria (Solomons et al., 2015). MZN was not considered as evidence of microbiological confirmation given sub-optimal specificity and the possibility of false positives (Wang et al., 2016; Heemskerk et al., 2018). Patients with confirmed viral, cryptococcal, or bacterial meningitis, intracranial tumor, intracranial hematoma, underlying malignancy, or HIV infection were excluded from the study (Figure 1). People living with HIV were excluded because there were only a small number representing a very specific subgroup with unique and well-described risk factors (Marais et al., 2011).

The modified Barthel Index assesses a person’s functional independence in daily activities and is useful to track neurological recovery over time (Collin et al., 1988). The Barthel Index consists of 10 items with a designated numerical value that corresponds to the level of assistance required to perform specific tasks. The total score ranges from 0 to 20, with higher scores indicating greater independence. We conducted telephonic outcome assessments, utilizing the modified Barthel Index (Supplementary Panel S2) (Collin et al., 1988), 9–24 months post treatment completion (Jha et al., 2015). Patients who were ‘lost to follow up’ after completing TBM treatment were excluded from comparative outcome analyses. A modified Bathel Index score of <12 was categorized as “poor outcome,” indicating diminished functional status. Poor outcome was only measured in those in whom an outcome was recorded. In general, TB treatment was only commenced after diagnostic work-up and collection of CSF samples, but in some instances, documentation was unclear.

All patients received standard WHO-recommended TBM treatment with isoniazid (300 mg/d), rifampicin (450–600 mg/d), pyrazinamide (20–30 mg/kg/d), and ethambutol (15–20 mg/kg/d) orally during the 2-month intensive phase (World Health Organization, 2010; Thwaites, 2013), with isoniazid and rifampicin during the 7–12 month continuation phase (Thwaites, 2013; Thwaites et al., 2013; Jullien et al., 2016; Falzon et al., 2017). Intravenous dexamethasone (0.4 mg/kg/day) or oral prednisone (40 mg/day) was given for severe disease at baseline (judged by the treating clinician), if a patient’s condition worsened after the start of treatment (Gilpin et al., 2018; Mirzayev et al., 2021). Additional adjuvant treatments, including mannitol, hypertonic saline, acetazolamide, and external CSF drainage or ventriculoperitoneal shunt, were used as clinically indicated. A combination of rifampicin (600 mg/kg/d) and levofloxacin (500–1,000 mg/d) or moxifloxacin (400–800 mg/d) was given intravenously if patients were unable to take oral medication or worsened on treatment. Patients with drug-resistant TBM received standard treatment since drug susceptibility testing (DST) was conducted retrospectively. Response to treatment was assessed by clinical examination during treatment and telephone follow-up 9–24 months after treatment completion.

### Specimen collection and testing

CSF was collected for routine, biochemical, cytological, and microbiological analyses. CSF protein and glucose were determined by immunoturbidimetry (Beckman Coulter DXA5000). CSF cell counts included total white blood cells, lymphocytes, neutrophils, and monocytes. CSF microscopy and MZN staining were performed using 0.5 mL of CSF loaded into a cytospin chamber with poly-lysine-coated slides and centrifuged at 70×*g* for 5 min, according to a standard protocol (Wang et al., 2016). The slide was fixed with 4% paraformaldehyde for 15 min at room temperature and sent to Xijing Hospital of the Fourth Military Medical University for reading. They were permeabilized with 0.3% TritonX-100 for 30 min, before staining with carbolfuchsin containing 0.3% TritonX-100 and counterstained with methylene blue. All slides stained by the modified method were observed under oil immersion at a magnification of 1,000 (Chen et al., 2012). Three hundred fields on each slide were examined documenting the number of fields in which acid-fast bacilli and their intracellular or extracellular location were observed. All positive slides were confirmed by an experienced technician, and 25% of slides were selected for random quality assurance review (Feng et al., 2014; Wang et al., 2016).

A WHO-approved NAAT (Xpert MTB/RIF, Cepheid, Sunnyvale, CA, United States) (Boehme et al., 2010) or *M. tuberculosis* culture (BACTEC MGIT 960, Bio-Rad Laboratories, Hercules, CA, United States) using standard operating procedures (Krüüner et al., 2006) was performed on all patients. At least 2 mL of CSF was collected. This was first sent to Xijing Hospital where MGIT 960 culture was performed on all specimens. Xpert MTB/RIF was performed at the same time if locally available, but there were periods of interruption when the test was unavailable or unfunded. Remaining CSF and cultured strains were stored at −80°C before transfer to the National Tuberculosis Reference Laboratory of the Chinese Center for Disease Control and Prevention (CDC) in Beijing, where batch Xpert MTB/RIF testing was performed on frozen CSF specimens if an adequate volume was available, and no previous Xpert MTB/RIF test result recorded. Phenotypic DST, as well as gene sequencing and spoligotyping were performed on all viable strains if an adequate amount of DNA could be harvested. Batched phenotypic DST was performed for isoniazid (H), rifampin (R), ethambutol (E), streptomycin (S), kanamycin (K), amikacin (A), capreomycin (C), moxifloxacin (Mfx), levofloxacin (Lfx), para-aminosalicylic acid (PAS), and prothionamide (Pto) on solid LJ medium, according to standard protocols (World Health Organization, 2011). Following resistance detection, the MGIT 960 system (Krüüner et al., 2006) was used to determine the mean inhibitory drug concentration (MIC) (Woods et al., 2011). DNA was extracted from freshly cultured colonies on LJ medium and processed using standard methodology for gene sequencing and spoligotyping (Gori et al., 2005). Strains were identified by *16–23467, rrs1690, and 16 s555* gene sequencing (Supplementary Panel S3) (Clarridge, 2004), compared with the *M. tuberculosis* reference strain H37Rv, and deposited in GenBank^1^ (Wang et al., 2016); whole genome sequencing was not performed. Genotypic DST was performed using standard primers for the *inhA, katG, rpoB, embB, gyrA, gyrB, rrs-KAN, eis, rpsL,* and *gidB* genes (Supplementary Panel S4) (Maus et al., 2005; Avalos et al., 2015; Cohen et al., 2015).

### Statistical analyses

Continuous variables (age, TBM score, leukocytes, lymphocytes, neutrophils, monocytes, protein, glucose, intracranial pressure, and Barthel score) were expressed as the median and interquartile range (IQR). Categorical variables (female sex, fever, headache, vomiting, neck stiffness, seizures, BMRC grade, CXR suggestive of active cavitating disease or disseminated/miliary TB, reported diabetes, hydrocephalus, infarcts, basal meningeal enhancement, granulomas/tuberculomas, any drug resistance detected, Beijing genotype, TBM treatment completed, lost to follow-up, death, and poor outcome) were expressed as counts and proportions. Differences between microbiologically confirmed TBM cases that had a TBM score of ≥12 and < 12 were assessed by the χ^2^ test for categorical variables, and the Mann–Whitney U test was performed to assess continuous variables. Continuous variables were also assessed using the Kruskal–Wallis test (non-parametric one-way analysis of variance, ANOVA), and categorical variables were assessed using the χ^2^ test, with Bonferroni adjustments. Multivariate logistic regression was used to analyze risk factors for poor outcome, including all factors significantly associated (*p* < 0.05) with univariate analyses. Comparative results were presented as odds ratios (ORs) with 95% confidence interval (CI). Receiver operating characteristic (ROC) curves were constructed to assess diagnostic accuracy. All statistical analyses were performed using statistical package for social sciences (SPSS) version 20.0 and GraphPad Prism 7.0. Instances with missing data (*n* = 42) were excluded from the particular analysis, instead of imputing missing values.

## Results

**Figure 2:**
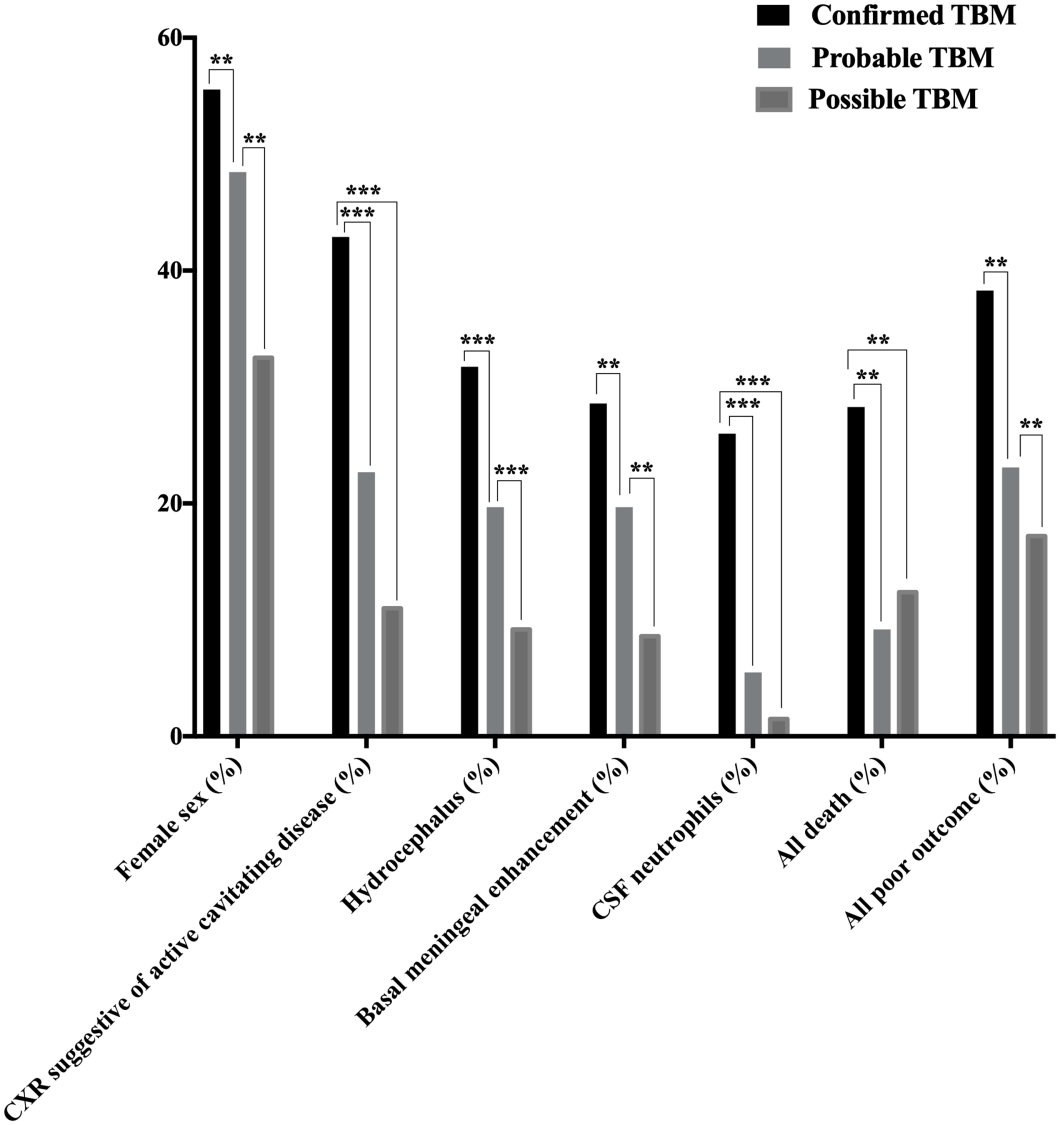
Disease characteristics observed among patients with confirmed, probable, and possible TBM^a^. TBM, tuberculous meningitis; CSF, cerebrospinal fluid; CXR, chest X-ray; TB, tuberculosis. Female sex, CXR suggestive of active cavitating disease, hydrocephalus, basal meningeal enhancement, CSF neutrophils, all death^b^, and all poor outcome^c^ were significantly associated with confirmed TBM compared with probable or possible TBM. *p < 0.05, **p < 0.01, and ***p < 0.001. ^a^According to the consensus uniform research case definition criteria (Marais et al., 2010) (Supplementary Panel S1). ^b^Death during or after treatment; assessed 9–24 months after treatment completion (Supplementary Panel S2) (Collin et al., 1988). ^c^Death during or after treatment or Barthel index score < 12; assessed 9–24 months after treatment completion (Supplementary Panel S2) (Collin et al., 1988). “Confirmed TBM” required a positive *M. tuberculosis* culture or Xpert MTB/RIF on CSF. “Probable TBM” required a TBM score of ≥12 given that all patients received brain imaging, while patients with a TBM score of 6–11 was classified as “possible TBM”.

A total of 341 presumptive TBM patients met selection criteria; 63 confirmed TBM (25 culture positive, 42 Xpert-MTB/RIF positive), 66 probable TBM, 163 possible TBM, and 49 “not TBM” according to uniform research TBM case definition criteria (Marais et al., 2010) (Figure 1). Among 292 patients started on TBM treatment, 93.1% (270/292) had an outcome reported and were included in comparative analyses. In total, 83.3% (50/60) confirmed TBM, 64.6% (42/65) probable TBM, and 33.1% (48/145) possible TBM patients completed TBM treatment. The 49 patients with presumptive TBM who were excluded had an alternative cause (9 viral meningitis, 19 cryptococcal meningitis, 10 bacterial meningitis, 7 intracranial tumor, 1 intracranial hematoma, an 1 acute leukemia) identified. Table 1 provides an overview of the TBM cohort comparing patients with confirmed, probable, and possible TBM. Key differences are shown in Figure 2. Female sex, CXR suggestive of active cavitating disease, hydrocephalus, basal meningeal enhancement, CSF neutrophils, all death, and all poor outcomes were significantly associated with confirmed TBM compared with probable or possible TBM.

**Table 1:** Comparison of baseline characteristics between confirmed, probable, and possible TBM patients.

Characteristic	Confirmed TBM^a^, *N* = 63(%)	Probable TBM^b^, *N* = 66(%)	Possible TBM^c^, *N* = 163(%)	*p*	Multiple comparisons
Median age-years (IQR)	26 (3–82)	34.5(2–64)	29 (1–80)	0.513	–
Female sex	35 (55.6)	32 (48.5)	53 (32.5)	**0.003**	**a > b > c**
Median TBM score (IQR)^d^	14 (6–20)	13 (12–19)	8 (6–11)	**<0.001**	**a = b > c**
Reported diabetes	1 (1.6)	2 (3.0)	1 (0.6)	0.357	–
History					
Fever	43 (68.3)	54 (81.8)	113 (69.3)	0.125	–
Headache	48 (76.2)	56 (84.9)	109 (66.9)	**0.017**	**b > a > c**
Vomiting	27 (42.9)	39 (59.1)	85 (52.2)	0.180	–
Neck stiffness	24 (38.1)	45 (68.2)	95 (58.3)	**0.002**	**a < b = c**
Seizures	6 (9.5)	10 (15.2)	27 (16.6)	0.405	–
BMRC grading^e^					
I	32 (50.8)	23 (34.9)	72 (44.2)	0.182	–
II	21 (33.3)	24 (36.4)	67 (41.1)	0.521	–
III	10 (15.9)	19 (28.8)	24 (14.7)	**0.038**	**b > a > c**
Imaging					
CXR suggestive of active cavitating disease	27 (42.9)	15 (22.7)	18 (11.0)	**<0.001**	**a > b = c**
CXR indicative of disseminated/miliary TB	6 (9.5)	8 (12.1)	1 (0.6)	**<0.001**	**a = b > c**
Hydrocephalus	20 (31.8)	13 (19.7)	15 (9.20)	**<0.001**	**a > b > c**
Infarcts	15 (23.8)	17 (25.8)	17 (10.4)	**0.005**	**a = b > c**
Basal meningeal enhancement	18 (28.6)	13 (19.7)	14 (8.6)	**0.001**	**a > b > c**
Granulomas/tuberculomas	1 (1.6)	1 (1.5)	0	0.280	–
CSF findings					
Median leukocyte count—cells/𝜇L (IQR)	141.5 (0–6,100)	94.0 (0–1,355)	96.0 (0–5,450)	0.360	–
Leukocyte (50–500)—cells/𝜇L	43 (68.3)	42 (63.6)	85 (52.2)	0.053	–
Median lymphocytes—% (IQR)	60.8 (1.0–98.0)	77.0 (3.5–98.5)	74.0 (2.0–99.0)	0.053	–
Lymphocytes >50 (%)	38 (60.3)	52 (78.8)	112 (68.7)	0.074	–
Median neutrophils—% (IQR)	26.0 (0–94.5)	5.5 (0–95)	1.5 (0–94)	**<0.001**	**a > b = c**
Median monocytes—% (IQR)	8.5 (0–53)	7.5 (0–47)	10.5 (0–85)	**0.014**	**b < c**
Median protein—mg/dL (IQR)	1.5 (0.2–7.4)	1.3 (0.07–9.3)	0.8 (0.04–6.0)	**<0.001**	**a = b > c**
Protein >1.0 mg/dL	37 (58.7)	41 (62.1)	47 (28.8)	**<0.001**	**a = b > c**
Median glucose—mmol/L (IQR)	1.8 (0.4–4.5)	2.1 (0.4–5.2)	2.5 (0.03–6.2)	**<0.001**	**a = b < c**
Glucose <2.2 mmoL/L	37 (58.7)	38 (57.6)	36 (22.1)	**<0.001**	**a = b > c**
Median intracranial pressure (mmH_2_O) (IQR)	260 (100–400)	240 (60–600)	190 (60–400)	**<0.001**	**a = b > c**
Intracranial hypertension (>180 mmH_2_O)^f^	43 (68.3)	47 (71.2)	76 (46.6)	**<0.001**	**a = b > c**
Outcome	N = 60 (95.2)	N = 65 (98.5)	N = 145 (89.0)	**0.030**	–
TBM treatment completed^g^	50 (83.3)	42 (64.6)	48 (33.1)	**<0.001**	**a = b > c**
Lost to follow up	3 (5.0)	1 (1.5)	18 (12.4)	**0.017**	–
Death (on treatment)	5 (8.3)	5 (7.7)	16 (11.0)	0.696	–
Death (post treatment)^h^	12 (20.0)	1 (1.5)	2 (1.4)	**<0.001**	**a > b = c**
All death	17 (28.3)	6 (9.2)	18 (12.4)	**0.005**	**a > b = c**
Alive with poor outcome^i^	6 (10.0)	9 (13.8)	7 (4.8)	0.073	–
Median Barthel score (IQR)^j^	14 (2–20)	14 (2–20)	16 (2–20)	**<0.001**	**a = b < c**
All Poor outcome^k^	23 (38.3)	15 (23.1)	25 (17.2)	**0.005**	**a > b > c**

CXR, chest X-ray; TBM, tuberculous meningitis; TB, tuberculosis; CSF, cerebrospinal fluid; BMRC, British Medical Research Council; IQR, interquartile range.

Continuous variables are presented as median (interquartile range), and categorical variables are presented as counts (proportions). The Bonferroni method was used to adjust the significance level to perform multiple testing. If statistically significant, continuous variables were analyzed by the Kruskal–Wallis test followed by post-hoc analysis with Bonferroni adjustment to compare differences between confirmed TBM, probable TBM, and possible TBM. Categorical parameters were analyzed using χ^2^ test with Bonferroni adjustment for multiple testing. *p*-values < 0.05 were considered statistically significant (shown with bold).

a Confirmed TBM defined by a positive M. tuberculosis culture or Xpert MTB/RIF on CSF (Marais et al., 2010).

b Probable TBM required a TBM score of ≥12 given that all patients received brain imaging (3).

c Patients with a TBM score of 6–11 was classified as possible TBM (3).

d According to the consensus uniform research case definition criteria (Marais et al., 2010) (see Supplementary Panel S1).

e TBM severity grade according to the revised British Medical Research Council disease severity grade (BMRC 1948) with stage 3 being most severe (Solomons et al., 2015).

f Normal intracranial pressure typically ranges between 80 and 180 mmH_2_O. Values exceeding 180 mmH_2_O are indicative of intracranial hypertension (Gomez-Beldarrain and García-Moncó, 2018; Wang et al., 2023).

g Patients were treated for TBM for 9–12 months.

h Assessed 9–24 months after treatment completion.

i Modified Barthel Index score < 12 (excluding dead); assessed 9–24 months after treatment completion.

j See Supplementary Panel S2 for calculation of Modified Barthel Index score (excluding dead) (Collin et al., 1988).

k Death during or after treatment or Barthel index score < 12; assessed 9–24 months after treatment completion. Only reported for those in whom an outcome was reported; lost to follow-up excluded.

Table 2 compares the baseline characteristics of patients with confirmed TBM (culture and/or Xpert MTB/RIF positive) with those who were MZN-positive, but culture and Xpert-MTB/RIF-negative, on CSF. A chest X-ray suggestive of active cavitating disease, hydrocephalus, basal meningeal enhancement, CSF protein ≥1.4 mg/dL, CSF glucose <2.2 mmol/L, TBM score ≥ 12, and all poor outcomes was significantly associated with confirmed TBM compared with MZN staining-positive but culture and Xpert MTB/RIF-negative cases. Nearly one-third (17 of 63; 27.0%) of patients with confirmed TBM had a TBM score of <12. Since no patients with a TBM score of <6 were included in the study, we can only compare those with a score of ≥12 and 6–11. Supplementary Table S1 compares the characteristics of patients with confirmed TBM who had a TBM score of ≥12 and 6–11. Among confirmed TBM patients, those with a TBM score of <12 exhibited less classic meningitis symptoms and signs and had better outcomes compared with those with a TBM score of ≥12. Interestingly, two patients (1 male of 23 years with fever, headache, vomiting, and reduced consciousness and 1 female of 47 years without any classic meningitis symptoms) with confirmed TBM (both CSF Xpert MTB/RIF positive, but culture-negative) had pristine CSF (total leukocyte count <1 × 10^6^ cells/L, normal protein, and glucose levels) and normal CNS imaging. One had a good outcome without completing TBM treatment and one was lost to follow up.

**Table 2:** Comparison of baseline characteristics in patients with confirmed TBM^a^ and those that were MZN staining-positive but culture and Xpert-MTB/RIF-negative on cerebrospinal fluid.

Characteristic	Confirmed TBM (culture or Xpert positive)	MZN positive; culture and Xpert negative	*p*
	*N* = 63 (%)	*N* = 130 (%)	
Median age-years (IQR)	26 (3–82)	33 (1–74)	0.263
Female sex	35 (55.6)	58 (44.6)	0.154
Median TBM score (IQR)^b^	14 (6–20)	10 (6–19)	**<0.001**
TBM score ≥ 12	46 (73.0)	57 (43.8)	**<0.001**
Reported diabetes	1 (1.6)	2 (1.5)	0.979
History			
Fever	43 (68.3)	98 (75.4)	0.295
Headache	48 (76.2)	98 (75.4)	0.903
Vomiting	27 (42.9)	72 (55.4)	0.103
Neck stiffness	24 (38.1)	81 (62.3)	**0.002**
Seizures	6 (9.5)	14 (10.8)	0.790
BMRC grading^c^			
I	32 (50.8)	52 (40.0)	0.156
II	21 (33.3)	52 (40.0)	0.371
III	10 (15.9)	26 (20.0)	0.490
Imaging			
CXR suggestive of active cavitating disease	27 (42.9)	18 (13.8)	**<0.001**
CXR indicative of disseminated/miliary TB	6 (9.5)	8 (6.2)	0.415
Hydrocephalus	20 (31.7)	20 (15.4)	**0.009**
Infarcts	15 (23.8)	22 (16.9)	0.254
Basal meningeal enhancement	18 (28.6)	17 (13.1)	**0.009**
Granulomas/tuberculomas	1 (1.6)	1 (0.8)	0.599
CSF findings			
Median total leukocyte count—cells/𝜇L (IQR)	141.5 (0–6,100)	110 (0–5,450)	0.857
Median lymphocytes—% (IQR)	60.8 (1.0–98.0)	69.8 (2.0–98.5)	0.272
Median neutrophils—% (IQR)	26.0 (0–94.5)	7.3 (0–95.0)	0.093
Median monocytes—% (IQR)	8.5 (0–53.0)	8.3 (0–62.5)	0.896
Median protein—mg/dL (IQR)	1.5 (0.2–7.4)	1.0 (0.04–9.3)	**0.029**
Protein ≥1.4 mg/dL	33 (52.4)	41 (31.5)	**0.005**
Median glucose—mmol/L (IQR)	1.8 (0.4–4.5)	2.3 (0.3–6.2)	**0.012**
Glucose <2.2 mmoL/L	37 (58.7)	56 (43.1)	**0.041**
Median intracranial pressure—mmH_2_O (IQR)	260 (100–400)	210 (60–600)	**0.001**
Intracranial hypertension (>180 mmH_2_O)^d^	43 (68.3)	75 (57.7)	0.158
Outcome	N = 60 (95.2)	N = 123 (94.6)	0.855
TBM treatment completed^e^	50 (83.3)	63 (51.2)	**<0.001**
Lost to follow-up	3 (5.0)	7 (5.7)	0.847
Death (on treatment)	5 (8.3)	11 (8.9)	0.891
Death (post treatment)^f^	12 (20.0)	2 (1.6)	**<0.001**
Alive with poor outcome^g^	6 (10.0)	12 (9.8)	0.959
Median Barthel score (IQR)^h^	14 (2–20)	16 (2–20)	**<0.001**
All poor outcome^i^	23 (38.3)	25 (20.3)	**0.009**

CXR, chest X-ray; TBM, tuberculous meningitis; MZN, modified Ziehl–Neelsen staining; TB, tuberculosis; CSF, cerebrospinal fluid; BMRC, British Medical Research Council; IQR, interquartile range.

Continuous variables are presented as median (IQR), and categorical variables are presented as counts (proportions). Differences were assessed by the χ^2^-test for categorical variables and the Mann-Whitney U test for continuous variables. *p*-values <0.05 were considered statistically significant (shown with bold).

a Confirmed TBM defined by a positive M. tuberculosis culture or Xpert MTB/RIF on CSF (Marais et al., 2010).

b According to consensus uniform research case definition criteria (Marais et al., 2010) (see Supplementary Panel S1).

c TBM severity grade according to the revised British Medical Research Council disease severity grade (BMRC 1948) with stage 3 being most severe (Solomons et al., 2015).

d Normal intracranial pressure typically ranges between 80 and 180 mmH_2_O. Values exceeding 180 mmH_2_O are indicative of intracranial hypertension (Gomez-Beldarrain and García-Moncó, 2018; Wang et al., 2023).

e Patients were treated for TBM for 9–12 months.

f Assessed 9–24 months after treatment completion.

g Modified Barthel Index score < 12 (excluding dead); assessed 9–24 months after treatment completion.

h See Supplementary Panel S2 for calculation of Modified Barthel Index score (excluding dead) (Collin et al., 1988).

i Death during or after treatment or Barthel index score < 12; assessed 9–24 months after treatment completion. Only reported for those in whom an outcome was reported; lost to follow-up excluded.

**Figure 3:**
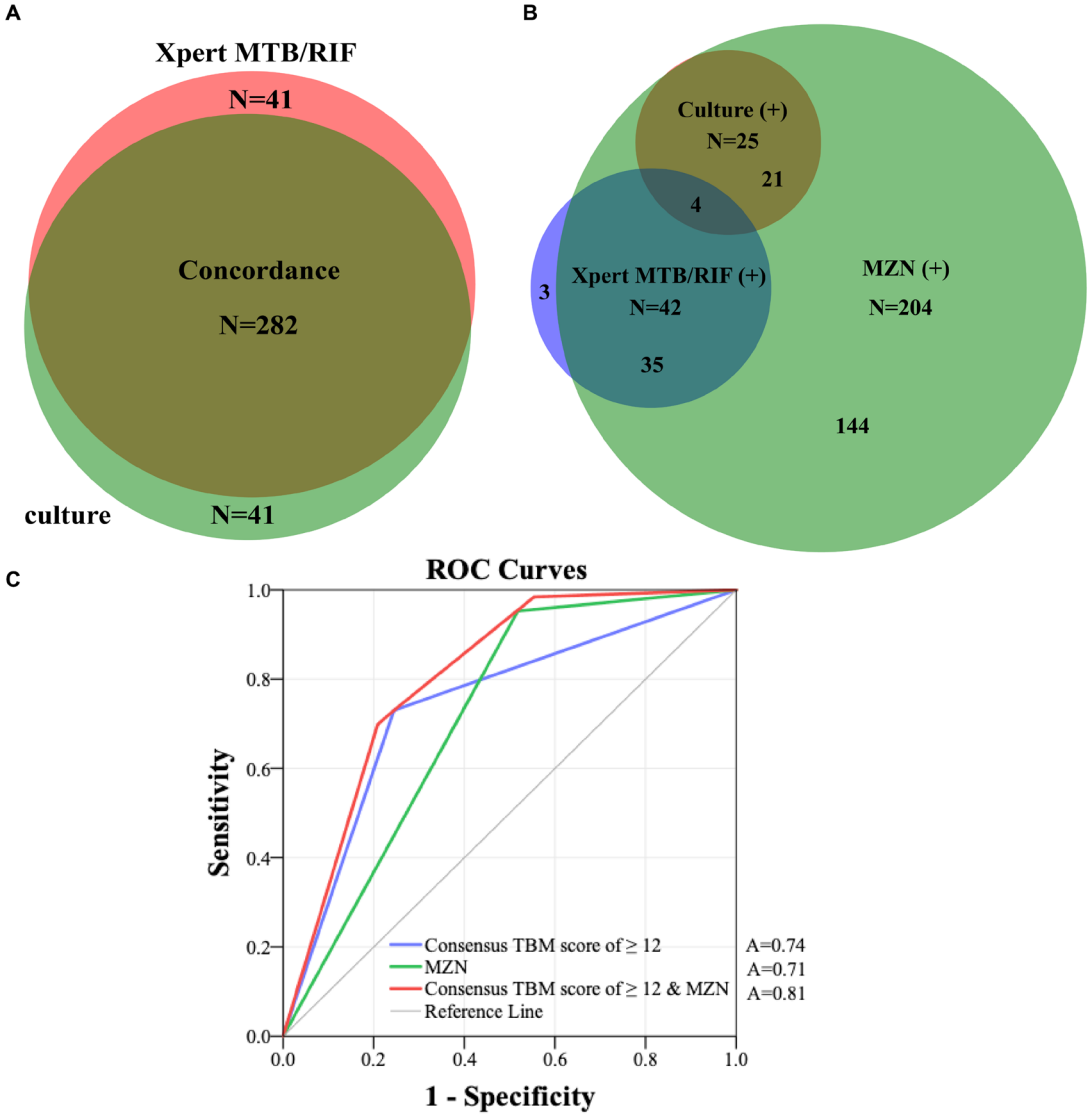
**(A)** Concordance of all CSF Xpert MTB/RIF and culture results in patients who had both tests performed. **(B)** The overlap of positive culture, Xpert-MTB/RIF and MZN results. **(C)** ROC curves of consensus TBM score of ≥12 and MZN with confirmed TBM as the reference standard in 341 presumptive TBM patients who had at least a CSF culture or Xpert MTB/RIF performed. MZN, modified Ziehl–Neelsen staining; TBM, tuberculous meningitis; CSF, cerebrospinal fluid; A, area under the curve; ROC, receiver operating characteristic.

All 341 presumed TBM patients underwent CSF culture, and 323 underwent Xpert-MTB/RIF testing. Figure 3A reflects the concordance of CSF culture and Xpert-MTB/RIF results (282 of 323; 87.3%), among all patients who underwent both tests. Figure 3B shows the overlap of positive culture and Xpert-MTB/RIF and MZN results. Among 63 confirmed TBM patients, 21 (33.3%) were culture-positive and Xpert-MTB/RIF-negative, while 38 (60.3%) were Xpert-MTB/RIF-positive and culture-negative. Among those who had both tests performed at the same time, 57.1% (4/7) culture-positive CSF specimens tested Xpert MTB/RIF-positive compared with zero (0/18) when tests were performed more than 30 days apart. While 95.2% (60/63) of confirmed TBM cases were MZN-positive, only 29.3% (60 of 204) of MZN-positive cases were culture or Xpert MTB/RIF-positive. Using data from all 341 presumptive TBM cases, with confirmed TBM as the reference standard, the ROC area under the curve (AUC) for MZN was 0.71 (95% CI: 0.66–0.78) with diagnostic sensitivity of 95.2% and specificity of 48.2%. The AUC for TBM score ≥ 12 was 0.74 (95% CI: 0.67–0.81) with diagnostic sensitivity of 73.0% and specificity of 75.5%. The AUC for TBM score ≥ 12 and positive MZN combined using binary logistic prediction was 0.81 (95%CI: 0.76–0.86), with diagnostic sensitivity of 70.0% and specificity of 79.1% (Figure 3C and Supplementary Table S2).

Table 3 reflects the multivariable logistic regression analysis of risk factors associated with poor outcome (including death) in 270 patients treated for TBM. BMRC grade III (OR = 4.369; 95%CI: 1.949–9.792) and hydrocephalus (OR = 2.520; 95%CI: 1.044–6.084) were associated with poor outcome, while BMRC grade III (OR = 5.172; 95%CI: 2.298–11.641), TBM score ≥ 15 (OR = 3.843; 95%CI: 1.372–10.761), age > 60 years (OR = 3.566; 95%CI: 1.022–12.442), and CSF neutrophil ratio ≥ 25% (OR = 2.298; 95%CI: 1.027–5.139) were most strongly associated with death (Table 4). Among those with confirmed TBM, CXR signs indicating disseminated/miliary TB (OR = 19.183; 95%CI: 1.601–229.896) was mostly associated with poor outcome (Supplementary Table S3). Moreover, CXR signs indicating disseminated/miliary TB (OR = 12.427; 95%CI: 1.138–135.758) and a higher TBM score (≥15, OR = 8.437; 95%CI: 1.328–53.585) were most strongly associated with death (Supplementary Table S4). In addition, BMRC grade III (OR = 5.129; 95%CI: 2.152–12.222), which essentially reflects a depressed level of consciousness, was strongly associated with poor outcome in probable and possible TBM cases (Supplementary Table S5). However, detected drug resistance was not associated with poor outcome or death in those with confirmed TBM (Supplementary Tables S3, S4).

**Table 3:** Multivariable logistic regression analysis for risk factors of poor outcome^a^ in 270 patients treated for TBM^b^.

Characteristic	Univariate	Multivariate model		
	OR (95%CI)	*p*	Adjusted OR (95% CI)	*p*
Neck stiffness	**2.146 (1.072–4.294)**	**0.031**	–	0.396
Seizures	**2.502 (1.252–4.998)**	**0.009**	–	0.072
BMRC grading^c^				
I	**0.214 (0.106–0.432)**	**<0.001**	**0.332 (0.136–0.810)**	**0.015**
III	**6.307 (3.238–12.283)**	**<0.001**	**4.369 (1.949–9.792)**	**<0.001**
TBM score^d^ ≥ 12	**2.558 (1.437–4.552)**	**0.001**	**–**	0.193
TBM score^d^ ≥ 15	**3.657 (1.763–7.584)**	**<0.001**	**–**	0.623
Imaging				
Hydrocephalus	**3.735 (1.928–7.237)**	**<0.001**	**2.520 (1.044–6.084)**	**0.040**
Basal meningeal enhancement	**3.006 (1.527–5.917)**	**0.001**	**–**	0.618
CXR suggestive of active cavitating disease	**–**	0.729	**–**	**–**
CXR indicative of disseminated/miliary TB	**–**	0.125	**–**	**–**
CSF findings				
Total leukocyte count >100 (cells/𝜇L)	**–**	0.798	**–**	**–**
Lymphocytes (%)	**–**	0.144	**–**	**–**
Neutrophils (%)	**–**	0.143	**–**	**–**
Glucose <2.2 mmoL/L	**1.819 (1.030–3.213)**	**0.039**	**–**	0.404
Protein >1 mg/dL	**–**	0.810	**–**	**–**
Intracranial pressure (mmH_2_O)	**1.004 (1.001–1.007)**	**0.009**	**–**	**–**
Intracranial hypertension (>180mmH_2_O)^e^	**1.942 (1.014–3.719)**	**0.045**		0.319

CXR, chest X-ray; TBM, tuberculous meningitis; OR, odds ratio; CI, confidence interval; BMRC grade, British Medical Research Council disease severity grade; CSF, cerebrospinal fluid; TB, tuberculosis. Age, Female sex, BMRC grade II, fever, headache, vomiting, CXR suggestive of active cavitating disease or disseminated/miliary TB, infarcts, granulomas/tuberculomas, CSF total leukocyte count, lymphocytes, monocytes, neutrophils, and protein were non-significant controlled covariates. *p*-values <0.05 were considered statistically significant (shown with bold).

a Death during or after treatment or Barthel index score < 12; assessed 9–24 months after treatment completion (see Supplementary Panel S2) (Collin et al., 1988).

b Among 292 patients started on TBM treatment, 270 patients followed for outcome (22 patients lost to follow-up) and included in comparative analyses.

c TBM severity grade according to the revised British Medical Research Council disease severity grade (BMRC 1948) with stage 3 being most severe (Solomons et al., 2015).

d According to the consensus uniform research case definition criteria (Marais et al., 2010) (see Supplementary Panel S1).

e Normal intracranial pressure typically ranges between 80 and 180 mmH_2_O. Values exceeding 180 mmH_2_O are indicative of intracranial hypertension (Gomez-Beldarrain and García-Moncó, 2018; Wang et al., 2023).

**Table 4:** Multivariable logistic regression analysis of risk factors for death^a^ in 270 patients treated for TBM^b^.

Characteristic	Univariate		Multivariate model	
	OR (95%CI)	*p*	Adjusted OR (95% CI)	*p*
Headache	**0.389 (0.190–0.798)**	**0.010**	**0.378 (0.161–0.888)**	**0.026**
Seizures	**3.002 (1.401–6.432)**	**0.005**	**–**	0.058
Age (years)	**1.023 (1.004–1.043)**	**0.019**	**–**	**–**
<15	**–**	0.055	**–**	**–**
15–60	**0.374 (0.186–0.752)**	**0.006**	**–**	**–**
>60	**5.687 (1.938–16.693)**	**0.002**	**3.566 (1.022–12.442)**	**0.046**
Consensus TBM score^c^	**1.154 (1.042–1.277)**	**0.006**	**–**	**–**
≥15	**4.158 (1.894–9.129)**	**<0.001**	**3.843 (1.372–10.761)**	**0.010**
≥12	**–**	0.079	**–**	**–**
BMRC grading^d^				
III	**6.568 (3.179–13.571)**	**<0.001**	**5.172 (2.298–11.641)**	**<0.001**
Imaging				
Hydrocephalus	**2.573(1.216–5.447)**	**0.013**	**–**	0.598
Infarcts	1.572 (0.712–3.469)	0.263	**–**	**–**
Basal meningeal enhancement	**2.458 (1.141–5.293)**	**0.022**	**–**	0.752
CXR suggestive of active cavitating disease	**–**	0.442	**–**	**–**
CXR indicative of disseminated/miliary TB	**–**	0.212	**–**	**–**
CSF findings				
Neutrophils (%)	**1.011 (1.001–1.022)**	**0.034**	**–**	**–**
Neutrophils ≥25 (%)	**3.049 (1.529–6.080)**	**0.002**	**2.298 (1.027–5.139)**	**0.043**
Intracranial pressure—mmH_2_O	–	0.200	–	–

CXR, chest X-ray; OR, odds ratio; CI, confidence interval; BMRC grade, British Medical Research Council disease severity grade; TBM, tuberculous meningitis; CSF, cerebrospinal fluid; TB, tuberculosis. Female, fever, vomiting, neck stiffness, BMRC grading I, II, CXR suggestive of active cavitating disease or disseminated/miliary TB, granulomas/tuberculomas, CSF leukocyte, CSF lymphocyte, CSF monocytes, CSF protein, CSF glucose, and intracranial pressure were non-significant controlled covariates. *p*-values <0.05 were considered statistically significant (shown with bold).

a Death during or after treatment; assessed 9–24 months after treatment completion (see Supplementary Panel S2) (Collin et al., 1988).

b Among 292 patients started on TBM treatment, 270 patients followed for outcome (22 patients lost to follow-up) and included in the comparative analyses.

c According to the consensus uniform research case definition criteria (Marais et al., 2010) (see Supplementary Panel S1).

d TBM severity grade according to the revised British Medical Research Council disease severity grade (BMRC 1948) with stage 3 being most severe (Solomons et al., 2015).

Resistance to at least one TB drug was documented in nearly one-third (16 of 63; 25.4%) of confirmed TBM cases (Supplementary Table S1), and 50% (8 of 16) had rifampicin resistance identified by Xpert-MTB/RIF. Drug resistance was detected in 48.0% (12 of 25) of all cultured strains and 3 had multidrug-resistance (MDR; resistance to isoniazid and rifampicin). All four strains with phenotypic isoniazid resistance had high-level resistance with mutations in the *katG* gene (S315T, R463L). One case with phenotypic low-level rifampin resistance was not detected by Xpert MTB/RIF, and no mutations could be identified upon *rpoB* gene sequencing. Notably, there was no phenotypic fluoroquinolone resistance detected, despite the presence of non-synonymous mutations in the *gyrA* gene (Supplementary Table S6). All cultured strains belonged to *M. tuberculosis* complex, with the Beijing lineage being most common (20 of 25; 80%), including 80.0% (10 of 12) of all drug-resistant strains detected by culture.

## Discussion

This study represents the most comprehensive investigation of demographic, clinical, radiological, and laboratory descriptors associated with TBM diagnosis and outcome in Shaanxi province, China. The detailed description of prognostic factors in an HIV-uninfected population provides valuable new insight, especially in an Asian context. The fact that nearly one-third (27.0%) of confirmed TBM cases had a TBM score of <12, highlighting the difficulty of early accurate diagnosis and the shortcoming of traditional diagnostic methods. Our results indicate that a high TBM score (≥12) combined with a positive MZN result indicates reasonable diagnostic accuracy compared with a reference of confirmed TBM (as defined), but poor specificity remains a particular limitation of MZN (Wang et al., 2016; Heemskerk et al., 2018). A definitive CSF test with high sensitivity, such as Xpert-MTB/RIF Ultra (Cresswell et al., 2020) or culture (Hannan et al., 2010), should always be included in the diagnostic work-up. A study of 204 Ugandan adults with meningitis reported that the sensitivity of Xpert MTB/RIF Ultra was 76.5% (95% CI: 62.5–87.2) while that of Xpert MTB/RIF was 55.6% (44.0–70.4; *p* = 0.001 for the comparison of sensitivity between tests) compared with a reference standard of definite or probable TBM. The reduced sensitivity of Xpert MTB/RIF compared with Ultra is well established (Donovan et al., 2020) and also corresponds to our findings, where only 57.1% of culture-positive CSF specimens were Xpert MTB/RIF-positive if both tests were performed at the same time. Negative cultures in the presence of a positive Xpert MTB/RIF result might be related to low CSF bacillary load and sample processing that affected viability (Thuong et al., 2019). Samples were generally collected before TB treatment initiation, but this was not accurately recorded in all instances, and discrepancies may also represent a treatment effect.

Several studies served to highlight the key distinguishing features of TBM, including non-acute symptom onset (>5 days), low CSF leukocytes (<1,000 cells per mm^3^), elevated CSF protein (>100 mg/dL), and a low CSF: blood glucose ratio (<0.5) (Wilkinson et al., 2017). The association of a CSF glucose level of <2.2 mmol/L with confirmed TBM supports the findings from a UK study (Heemskerk et al., 2018), although comparative assessment of CSF and serum glucose is considered most informative (Solomons et al., 2016). Basal meningeal exudates, identified on contrast enhanced CT imaging, have been found to be highly specific for TBM and predictive of poor outcome (Bullock and Welchman, 1982). Although it was significantly associated with both confirmed TBM and probable TBM in our study, it was present in less than one-third of cases.

Unfortunately, TBM treatment outcomes remain poor outcome despite some recent treatment improvement (Méchaï and Bouchaud, 2019; Donovan et al., 2020; Huynh et al., 2022). In a study from Singapore, 38.9% (7 of 18) of TBM patients had a poor outcome (Modi et al., 2017), which was similar to a large series from India where 32.5% (165 of 507) had poor outcomes, 17.0% (86 of 507) died, and 15.6% (79 of 507) suffered from severe neurological sequelae (Erdem et al., 2015). In our study, 38.3% (23 of 60) of those with confirmed TBM had a poor outcome and 28.3% (17 of 60) died. Interestingly, only 31.3% (5 of 16) and 25% (4 of 16) of drug-resistant TBM patients died despite receiving suboptimal therapy. Similar to our findings TBM prognosis has been associated with old age, disease severity (Schoeman and Donald, 2013), hydrocephalus, disseminated/miliary TB (Gu et al., 2015), and a high neutrophil-to-lymphocyte ratio (Chan et al., 2003; Török, 2015; Li et al., 2017; Kamat et al., 2018; Gu et al., 2023). Detected drug resistance was not associated with poor outcome in our study, aligning with some past observations (Seddon et al., 2012), but in contrasts to studies where multidrug resistance (combined resistance to isoniazid and rifampicin) was strongly associated with mortality (Thwaites et al., 2005). This discrepancy could be attributed to the small number of drug-resistant strains, and the predominance of mono-resistant strains in our study.

In our study, Beijing lineage strains were predominated, broadly reflecting the percentage (990 of 1,189; 83.3%) of *M. tuberculosis* isolates obtained from pulmonary TB patients in another study from China (Liu et al., 2018) and in 71.1% (32 of 45) of isolates from relapsed TB cases in Singapore (Sun et al., 2006). These data is very different from India where only 8.9% (11/124) of isolates from North India (Mathuria et al., 2017), were identified as Beijing genotype and most strains were from lineage 3 (Singh et al., 2021). Beijing lineage strains have been associated with poor TB treatment outcome in some studies (Feng et al., 2008; Liu et al., 2020), but the association with TBM has been variable (Maree et al., 2007; Buu et al., 2010; Liu et al., 2018). Our analysis was limited by small sample size, but we could not demonstrate an association between Beijing genotype and TBM outcome.

It is important to acknowledge major study limitations. Retrospective data collection was conducted over an extended observation period, with risk of missing data and data inconsistency. These risks were minimized by comprehensive assessment of all clinical notes using a standard data collection template. We excluded those cases in whom baseline data was inadequate for accurate disease classification and those in whom outcomes could not be assessed. This excluded many patients and may have introduced selection bias, with the outcomes representative of those who received optimal care under the local circumstances. While adjunctive corticosteroid use is recommended by WHO guidelines (World Health Organization, 2010; Thwaites, 2013) during the initial 6–8 weeks of TBM treatment, there is currently no consensus on its routine use in China (Prasad et al., 2016; Brett et al., 2020; Huynh et al., 2022). Some clinicians argue that adjunctive corticosteroid use does not significantly improve the prognosis in mild cases (Schoeman et al., 1997), and that the risk of complications such as infection resulting from immune compromise, hyperglycemia, and stress ulcers (Török, 2015; Wilkinson et al., 2017) may outweigh the benefit. A Cochrane systematic review concluded that adjunctive corticosteroids reduce death from TBM by almost a quarter, but no effect on disabling neurological deficits could be demonstrated (Wilkinson et al., 2017; Huynh et al., 2022). In addition, due to variations in the LHA4 genotype, certain patients may experience worsening symptoms after corticosteroid administration (Donovan et al., 2018), and at present, we do not have a mechanism to identify this risk group.

Since MGIT 960 culture and Xpert MTB/RIF were not consistently performed at the same time and before TB treatment initiation, it is difficult to compare between diagnostic yield and accuracy. However, at least one confirmatory test (MGIT 960 culture or Xpert MTB/RIF) was performed in all cases included in the comparative analyses. Unfortunately, Xpert MTB/RIF Ultra^®^, which is the most sensitive NAAT for CSF diagnosis (Donovan et al., 2020), was not available at the time of the study. WHO first recommended the use of the Xpert MTB/RIF assay in the diagnosis of TB and extrapulmonary TB in 2013 (World Health Organization, 2014), while the Xpert-MTB/RIF Ultra only became available in 2017 (Chakravorty et al., 2017). Since our data collection period extended from May 2009 to April 2019, and in order to ensure internal consistency, we only performed the Xpert MTB-RIF test. Finally, since DST was only performed at the end of the study, these results could not inform patient management and treatment was empirical. Patients who did not improve on standard therapy were treated with high-dose rifampicin and moxifloxacin, which does not represent optimal treatment in cases with drug resistant TBM. In addition, we were unable to correlate HIV infection (excluded), hyponatremia, and other unmeasured risk factors potentially associated with prognosis. Despite these limitations, the data obtained from this study offer valuable insights into TBM clinical presentation and prognostic factors in China.

## Conclusion

TBM patients have variable presentations and those with a TBM score of <12 may be missed by traditional diagnostic approaches. Early TBM diagnosis remains challenging, but ready access to Xpert MTB/RIF Ultra should improve the accuracy of CSF testing. TBM patients that are older (>60 years) have higher TBM scores or CSF neutrophil ratios, have signs of disseminated/miliary TB, and are at greatest risk of death. In general, more effort needs to be done to improve early diagnosis and treatment outcome in TBM patients.

## Data availability statement

The original contributions presented in the study are publicly available. This data can be found in the NCBI BioProject repository (accession number PRJNA1069020) and in the article/Supplementary material.

## Ethics statement

The studies involving humans were approved by the Ethics Committee of Xijing Hospital of Air Force Medical University (Study No. KY20105255-1 and No. KY20163367-1) and the Ethics Committee of Guangzhou First People’s Hospital (Study No. K-2022-054-01). The studies were conducted in accordance with the local legislation and institutional requirements. Written informed consent for participation in this study was provided by the participants’ legal guardians/next of kin.

## Author contributions

TW: Conceptualization, Data curation, Formal analysis, Funding acquisition, Investigation, Methodology, Project administration, Resources, Software, Supervision, Validation, Visualization, Writing – original draft, Writing – review & editing. M-yL: Conceptualization, Data curation, Formal analysis, Funding acquisition, Writing – review & editing. X-SC: Data curation, Methodology, Resources, Supervision, Writing – review & editing. Q-sC: Conceptualization, Methodology, Supervision, Visualization, Writing – review & editing. ZL: Supervision, Validation, Visualization, Writing – review & editing. T-tL: Data curation, Supervision, Validation, Writing – review & editing. L-fZ: Data curation, Methodology, Supervision, Writing – review & editing. H-hW: Data curation, Methodology, Supervision, Writing – review & editing. G-dF: Data curation, Methodology, Supervision, Writing – review & editing. BM: Conceptualization, Data curation, Formal analysis, Investigation, Validation, Visualization, Writing – review & editing. GZ: Conceptualization, Data curation, Formal analysis, Funding acquisition, Investigation, Methodology, Project administration, Resources, Supervision, Validation, Visualization, Writing – review & editing.

## Footnotes

^1^ http://www.ncbi.nlm.nih.gov/BLAST/

## Glossary

MZN: modified Ziehl–Neelsen staining
TBM: tuberculous meningitis
CSF: cerebrospinal fluid
AUC: area under the curve
TB: tuberculosis
*M. tuberculosis*: *Mycobacterium tuberculosis*
MGIT: mycobacteria growth indicator tube
BMRC: British Medical Research Council
H: isoniazid
R: rifampin
E: ethambutol
S: streptomycin
K: kanamycin
A: amikacin
C: capreomycin
Mfx: moxifloxacin
Lfx: levofloxacin
PAS: para-aminosalicylic acid
Pto: prothionamide
DST: drug sensitivity test
HIV: human-immunodeficiency-virus
CXR: chest X-ray
OR: odds ratio
CI: confidence interval
SPSS: statistical package for social sciences

## References

[ref1] Arshad A. Dayal S. Gadhe R. Mawley A. Shin K. Tellez D.. (2020). Analysis of tuberculosis meningitis pathogenesis, diagnosis, and treatment. J. Clin. Med. 9:2962. doi: 10.3390/jcm9092962, PMID: 32937808 PMC7565176

[ref2] Avalos E. Catanzaro D. Catanzaro A. Ganiats T. Brodine S. Alcaraz J.. (2015). Frequency and geographic distribution of gyrA and gyrB mutations associated with fluoroquinolone resistance in clinical *Mycobacterium tuberculosis* isolates: a systematic review. PLoS One 10:e0120470. doi: 10.1371/journal.pone.0120470, PMID: 25816236 PMC4376704

[ref3] Boehme C. C. Nabeta P. Hillemann D. Nicol M. P. Shenai S. Krapp F.. (2010). Rapid molecular detection of tuberculosis and rifampin resistance. N. Engl. J. Med. 363, 1005–1015. doi: 10.1056/NEJMoa0907847, PMID: 20825313 PMC2947799

[ref4] Brett K. Dulong C. Severn M. (2020). Treatment of tuberculosis: a review of guidelines. Ottawa ON: Canadian Agency for Drugs and Technologies in Health. 33074600

[ref5] Bullock M. R. Welchman J. M. (1982). Diagnostic and prognostic features of tuberculous meningitis on CT scanning. J. Neurol. Neurosurg. Psychiatry 45, 1098–1101. doi: 10.1136/jnnp.45.12.1098, PMID: 6984460 PMC491690

[ref6] Buu T. N. Huyen M. N. van Soolingen D. Lan N. T. Quy H. T. Tiemersma E. W.. (2010). The *Mycobacterium tuberculosis* Beijing genotype does not affect tuberculosis treatment failure in Vietnam. Clin. Infect. Dis. 51, 879–886. doi: 10.1086/656410, PMID: 20836697

[ref7] Chakravorty S. Simmons A. M. Rowneki M. Parmar H. Cao Y. Ryan J.. (2017). The new Xpert MTB/RIF ultra: improving detection of mycobacterium tuberculosis and resistance to rifampin in an assay suitable for point-of-care testing. MBio 8:e00812-17. doi: 10.1128/mBio.00812-17, PMID: 28851844 PMC5574709

[ref8] Chan K. H. Cheung R. T. Fong C. Y. Tsang K. L. Mak W. Ho S. L. (2003). Clinical relevance of hydrocephalus as a presenting feature of tuberculous meningitis. QJM 96, 643–648. doi: 10.1093/qjmed/hcg108, PMID: 12925719

[ref9] Chen P. Shi M. Feng G. D. Liu J. Y. Wang B. J. Shi X. D.. (2012). A highly efficient Ziehl-Neelsen stain: identifying de novo intracellular mycobacterium tuberculosis and improving detection of extracellular *M. tuberculosis* in cerebrospinal fluid. J. Clin. Microbiol. 50, 1166–1170. doi: 10.1128/JCM.05756-11, PMID: 22238448 PMC3318527

[ref10] Clarridge J. E. 3rd. (2004). Impact of 16S rRNA gene sequence analysis for identification of bacteria on clinical microbiology and infectious diseases. Clin. Microbiol. Rev. 17, 840–862. doi: 10.1128/CMR.17.4.840-862.2004, PMID: 15489351 PMC523561

[ref11] Cohen K. A. Abeel T. Manson McGuire A. Desjardins C. A. Munsamy V. Shea T. P.. (2015). Evolution of extensively drug-resistant tuberculosis over four decades: whole genome sequencing and dating analysis of *Mycobacterium tuberculosis* isolates from KwaZulu-Natal. PLoS Med. 12:e1001880. doi: 10.1371/journal.pmed.1001880, PMID: 26418737 PMC4587932

[ref12] Collin C. Wade D. T. Davies S. Horne V. (1988). The Barthel ADL index: a reliability study. Int. Disabil. Stud. 10, 61–63. doi: 10.3109/09638288809164103 3403500

[ref13] Cresswell F. V. Tugume L. Bahr N. C. Kwizera R. Bangdiwala A. S. Musubire A. K.. (2020). Xpert MTB/RIF ultra for the diagnosis of HIV-associated tuberculous meningitis: a prospective validation study. Lancet Infect. Dis. 20, 308–317. doi: 10.1016/S1473-3099(19)30550-X 31924549 PMC7045085

[ref14] Donovan J. Cresswell F. V. Thuong N. T. T. Boulware D. R. Thwaites G. E. Bahr N. C. (2020). Xpert MTB/RIF ultra for the diagnosis of tuberculous meningitis: a small step forward. Clin. Infect. Dis. 71, 2002–2005. doi: 10.1093/cid/ciaa473, PMID: 32543658 PMC7643749

[ref15] Donovan J. Phu N. H. Thao L. T. P. Lan N. H. Mai N. T. H. Trang N. T. M.. (2018). Adjunctive dexamethasone for the treatment of HIV-uninfected adults with tuberculous meningitis stratified by leukotriene A4 hydrolase genotype (LAST ACT): study protocol for a randomised double blind placebo controlled non-inferiority trial. Wellcome Open Res. 3:32. doi: 10.12688/wellcomeopenres.14007.1, PMID: 30363837 PMC6182672

[ref16] Donovan J. Thwaites G. E. Huynh J. (2020). Tuberculous meningitis: where to from here? Curr. Opin. Infect. Dis. 33, 259–266. doi: 10.1097/QCO.0000000000000648, PMID: 32324614 PMC7259381

[ref17] Dorman S. E. Schumacher S. G. Alland D. Nabeta P. Armstrong D. T. King B.. (2018). Xpert MTB/RIF ultra for detection of mycobacterium tuberculosis and rifampicin resistance: a prospective multicentre diagnostic accuracy study. Lancet Infect. Dis. 18, 76–84. doi: 10.1016/S1473-3099(17)30691-6, PMID: 29198911 PMC6168783

[ref18] Erdem H. Ozturk-Engin D. Tireli H. Kilicoglu G. Defres S. Gulsun S.. (2015). Hamsi scoring in the prediction of unfavorable outcomes from tuberculous meningitis: results of Haydarpasa-II study. J. Neurol. 262, 890–898. doi: 10.1007/s00415-015-7651-5 25634680

[ref19] Falzon D. Schünemann H. J. Harausz E. González-Angulo L. Lienhardt C. Jaramillo E.. (2017). World Health Organization treatment guidelines for drug-resistant tuberculosis, 2016 update. Eur. Respir. J. 49:1602308. doi: 10.1183/13993003.02308-2016, PMID: 28331043 PMC5399349

[ref20] Feng G. D. Shi M. Ma L. Chen P. Wang B. J. Zhang M.. (2014). Diagnostic accuracy of intracellular *mycobacterium tuberculosis* detection for tuberculous meningitis. Am. J. Respir. Crit. Care Med. 189, 475–481. doi: 10.1164/rccm.201309-1686OC, PMID: 24450377 PMC3977721

[ref21] Feng J. Y. Su W. J. Tsai C. C. Chang S. C. (2008). Clinical impact of *Mycobacterium tuberculosis* W-Beijing genotype strain infection on aged patients in Taiwan. J. Clin. Microbiol. 46, 3127–3129. doi: 10.1128/JCM.01132-08, PMID: 18596137 PMC2546746

[ref22] Gilpin C. Korobitsyn A. Migliori G. B. Raviglione M. C. Weyer K. (2018). The World Health Organization standards for tuberculosis care and management. Eur. Respir. J. 51:1800098. doi: 10.1183/13993003.00098-2018 29567724

[ref23] Gomez-Beldarrain M. García-Moncó J. C. (2018). “Lumbar puncture and CSF analysis and interpretation” in CNS infections: a clinical approach. ed. García-Moncó J. C. (London: Springer-Verlag), 1–17.

[ref24] Gori A. Bandera A. Marchetti G. Degli Esposti A. Catozzi L. Nardi G. P.. (2005). Spoligotyping and *Mycobacterium tuberculosis*. Emerg. Infect. Dis. 11, 1242–1248. doi: 10.3201/eid1108.040982, PMID: 16102314 PMC3320497

[ref25] Gu Z. Liu B. Yu X. Cheng T. Han T. Tong L.. (2023). Association of blood neutrophil-lymphocyte ratio with short-term prognosis and severity of tuberculosis meningitis patients without HIV infection. BMC Infect. Dis. 23:449. doi: 10.1186/s12879-023-08438-y, PMID: 37407938 PMC10321014

[ref26] Gu J. Xiao H. Wu F. Ge Y. Ma J. Sun W. (2015). Prognostic factors of tuberculous meningitis: a single-center study. Int. J. Clin. Exp. Med. 8:4487-93., PMID: 26064373 PMC4443207

[ref27] Hannan A. Hafeez A. Chaudary S. Rashid M. (2010). Rapid confirmation of tuberculous meningitis in children by liquid culture media. J. Ayub Med. Coll. Abbottabad 22:171-5. 22455290

[ref28] Heemskerk A. D. Donovan J. Thu D. D. A. Marais S. Chaidir L. Dung V. T. M.. (2018). Improving the microbiological diagnosis of tuberculous meningitis: a prospective, international, multicentre comparison of conventional and modified Ziehl-Neelsen stain, GeneXpert, and culture of cerebrospinal fluid. J. Infect. 77, 509–515. doi: 10.1016/j.jinf.2018.09.003, PMID: 30217659 PMC6293313

[ref29] Ho J. Marais B. J. Gilbert G. L. Ralph A. P. (2013). Diagnosing tuberculous meningitis – have we made any progress? Trop. Med. Int. Health 18, 783–793. doi: 10.1111/tmi.12099 23521060

[ref30] Huynh J. Donovan J. Phu N. H. Nghia H. D. T. Thuong N. T. T. Thwaites G. E. (2022). Tuberculous meningitis: progress and remaining questions. Lancet Neurol. 21, 450–464. doi: 10.1016/S1474-4422(21)00435-X, PMID: 35429482

[ref31] Jha S. K. Garg R. K. Jain A. Malhotra H. S. Verma R. Sharma P. K. (2015). Definite (microbiologically confirmed) tuberculous meningitis: predictors and prognostic impact. Infection 43, 639–645. doi: 10.1007/s15010-015-0756-z, PMID: 25724799

[ref32] Jullien S. Ryan H. Modi M. Bhatia R. (2016). Six months therapy for tuberculous meningitis. Cochrane Database Syst. Rev. 9:CD012091. doi: 10.1002/14651858.CD012163 27581996 PMC5018659

[ref33] Kamat A. S. Gretschel A. Vlok A. J. Solomons R. (2018). CSF protein concentration associated with ventriculoperitoneal shunt obstruction in tuberculous meningitis. Int. J. Tuberc. Lung Dis. 22, 788–792. doi: 10.5588/ijtld.17.0008, PMID: 29914605

[ref34] Koh W. J. Ko Y. Kim C. K. Park K. S. Lee N. Y. (2012). Rapid diagnosis of tuberculosis and multidrug resistance using a MGIT 960 system. Ann. Lab. Med. 32, 264–269. doi: 10.3343/alm.2012.32.4.264, PMID: 22779067 PMC3384807

[ref35] Kohli M. Schiller I. Dendukuri N. Yao M. Dheda K. Denkinger C. M.. (2021). Xpert MTB/RIF ultra and Xpert MTB/RIF assays for extrapulmonary tuberculosis and rifampicin resistance in adults. Cochrane Database Syst. Rev. 1:CD012768. doi: 10.1002/14651858.CD012768.pub3 33448348 PMC8078545

[ref36] Krüüner A. Yates M. D. Drobniewski F. A. (2006). Evaluation of MGIT 960-based antimicrobial testing and determination of critical concentrations of first- and second-line antimicrobial drugs with drug-resistant clinical strains of *Mycobacterium tuberculosis*. J. Clin. Microbiol. 44, 811–818. doi: 10.1128/JCM.44.3.811-818.2006, PMID: 16517859 PMC1393078

[ref37] Li K. Tang H. Yang Y. Li Q. Zhou Y. Ren M.. (2017). Clinical features, long-term clinical outcomes, and prognostic factors of tuberculous meningitis in West China: a multivariate analysis of 154 adults. Expert Rev. Anti-Infect. Ther. 15, 629–635. doi: 10.1080/14787210.2017.1309974, PMID: 28343419

[ref38] Liu Q. Wang D. Martinez L. Lu P. Zhu L. Lu W.. (2020). *Mycobacterium tuberculosis* Beijing genotype strains and unfavourable treatment outcomes: a systematic review and meta-analysis. Clin. Microbiol. Infect. 26, 180–188. doi: 10.1016/j.cmi.2019.07.016, PMID: 31336202

[ref39] Liu H. Zhang Y. Liu Z. Liu J. Hauck Y. Liu J.. (2018). Associations between *Mycobacterium tuberculosis* Beijing genotype and drug resistance to four first-line drugs: a survey in China. Front. Med. 12, 92–97. doi: 10.1007/s11684-017-0610-z 29288283

[ref40] Liu Y. Zhang X. Zhang Y. Sun Y. Yao C. Wang W.. (2018). Characterization of *Mycobacterium tuberculosis* strains in Beijing, China: drug susceptibility phenotypes and Beijing genotype family transmission. BMC Infect. Dis. 18:658. doi: 10.1186/s12879-018-3578-7, PMID: 30547765 PMC6295058

[ref41] Marais S. Pepper D. J. Schutz C. Wilkinson R. J. Meintjes G. (2011). Presentation and outcome of tuberculous meningitis in a high HIV prevalence setting. PLoS One 6:e20077. doi: 10.1371/journal.pone.0020077, PMID: 21625509 PMC3098272

[ref42] Marais S. Thwaites G. Schoeman J. F. Török M. E. Misra U. K. Prasad K.. (2010). Tuberculous meningitis: a uniform case definition for use in clinical research. Lancet Infect. Dis. 10, 803–812. doi: 10.1016/S1473-3099(10)70138-9, PMID: 20822958

[ref43] Maree F. Hesseling A. C. Schaaf H. S. Marais B. J. Beyers N. van Helden P.. (2007). Absence of an association between *Mycobacterium tuberculosis* genotype and clinical features in children with tuberculous meningitis. Pediatr. Infect. Dis. J. 26, 13–18. doi: 10.1097/01.inf.0000247044.05140.c7, PMID: 17195699

[ref44] Mathuria J. P. Srivastava G. N. Sharma P. Mathuria B. L. Ojha S. Katoch V. M.. (2017). Prevalence of *Mycobacterium tuberculosis* Beijing genotype and its association with drug resistance in North India. J. Infect. Public Health 10, 409–414. doi: 10.1016/j.jiph.2016.06.007, PMID: 27496592

[ref45] Maus C. E. Plikaytis B. B. Shinnick T. M. (2005). Molecular analysis of cross-resistance to capreomycin, kanamycin, amikacin, and viomycin in *Mycobacterium tuberculosis*. Antimicrob. Agents Chemother. 49, 3192–3197. doi: 10.1128/AAC.49.8.3192-3197.2005, PMID: 16048924 PMC1196259

[ref46] Méchaï F. Bouchaud O. (2019). Tuberculous meningitis: challenges in diagnosis and management. Rev. Neurol. (Paris) 175, 451–457. doi: 10.1016/j.neurol.2019.07.007 31383464

[ref47] Mirzayev F. Viney K. Linh N. N. Gonzalez-Angulo L. Gegia M. Jaramillo E.. (2021). World Health Organization recommendations on the treatment of drug-resistant tuberculosis, 2020 update. Eur. Respir. J. 57:2003300. doi: 10.1183/13993003.03300-2020, PMID: 33243847 PMC8176349

[ref48] Modi M. Sharma K. Prabhakar S. Goyal M. K. Takkar A. Sharma N.. (2017). Clinical and radiological predictors of outcome in tubercular meningitis: a prospective study of 209 patients. Clin. Neurol. Neurosurg. 161, 29–34. doi: 10.1016/j.clineuro.2017.08.006, PMID: 28843114

[ref49] Prasad K. Singh M. B. Ryan H. (2016). Corticosteroids for managing tuberculous meningitis. Cochrane Database Syst. Rev. 4:CD002244. doi: 10.1002/14651858.CD002244.pub4 27121755 PMC4916936

[ref50] Schoeman J. F. Donald P. R. (2013). Tuberculous meningitis. Handb. Clin. Neurol. 112, 1135–1138. doi: 10.1016/B978-0-444-52910-7.00033-7 23622321

[ref51] Schoeman J. F. Van Zyl L. E. Laubscher J. A. Donald P. R. (1997). Effect of corticosteroids on intracranial pressure, computed tomographic findings, and clinical outcome in young children with tuberculous meningitis. Pediatrics 99, 226–231. doi: 10.1542/peds.99.2.226, PMID: 9024451

[ref52] Seddon J. A. Thwaites G. E. (2019). Tuberculous meningitis: new tools and new approaches required. Wellcome Open Res. 4:181. doi: 10.12688/wellcomeopenres.15591.1, PMID: 31803849 PMC6871354

[ref53] Seddon J. A. Visser D. H. Bartens M. Jordaan A. M. Victor T. C. van Furth A. M.. (2012). Impact of drug resistance on clinical outcome in children with tuberculous meningitis. Pediatr. Infect. Dis. J. 31, 711–716. doi: 10.1097/INF.0b013e318253acf8, PMID: 22411053

[ref54] Singh A. V. Singh S. Yadav A. Kushwah S. Yadav R. Sai D. K.. (2021). Genetic variability in multidrug-resistant *Mycobacterium tuberculosis* isolates from patients with pulmonary tuberculosis in North India. BMC Microbiol. 21:123. doi: 10.1186/s12866-021-02174-6, PMID: 33879047 PMC8059304

[ref55] Solomons R. S. Visser D. H. Donald P. R. Marais B. J. Schoeman J. F. van Furth A. M. (2015). The diagnostic value of cerebrospinal fluid chemistry results in childhood tuberculous meningitis. Childs Nerv. Syst. 31, 1335–1340. doi: 10.1007/s00381-015-2745-z, PMID: 25976864

[ref56] Solomons R. S. Visser D. H. Marais B. J. Schoeman J. F. van Furth A. M. (2016). Diagnostic accuracy of a uniform research case definition for TBM in children: a prospective study. Int. J. Tuberc. Lung Dis. 20, 903–908. doi: 10.5588/ijtld.15.0509, PMID: 27287642

[ref57] Sun Y. J. Lee A. S. Wong S. Y. Paton N. I. (2006). Association of *Mycobacterium tuberculosis* Beijing genotype with tuberculosis relapse in Singapore. Epidemiol. Infect. 134, 329–332. doi: 10.1017/S095026880500525X, PMID: 16207386 PMC2870406

[ref58] Tayyab N. Zaman G. Satti L. Ikram A. Gardezi A. H. Khadim M. T. (2018). Direct susceptibility testing on MGIT 960 TB system: a rapid method for detection of drug resistant tuberculosis. J. Coll. Physicians Surg. Pak. 28, 590–593. doi: 10.29271/jcpsp.2018.08.590, PMID: 30060785

[ref59] Thuong N. T. T. Vinh D. N. Hai H. T. Thu D. D. A. Nhat L. T. H. Heemskerk D.. (2019). Pretreatment cerebrospinal fluid bacterial load correlates with inflammatory response and predicts neurological events during tuberculous meningitis treatment. J. Infect. Dis. 219, 986–995. doi: 10.1093/infdis/jiy588, PMID: 30299487 PMC6386814

[ref60] Thwaites G. E. (2013). Advances in the diagnosis and treatment of tuberculous meningitis. Curr. Opin. Neurol. 26, 295–300. doi: 10.1097/WCO.0b013e3283602814 23493162

[ref61] Thwaites G. E. Lan N. T. Dung N. H. Quy H. T. Oanh D. T. Thoa N. T.. (2005). Effect of antituberculosis drug resistance on response to treatment and outcome in adults with tuberculous meningitis. J. Infect. Dis. 192, 79–88. doi: 10.1086/430616, PMID: 15942897

[ref62] Thwaites G. E. van Toorn R. Schoeman J. (2013). Tuberculous meningitis: more questions, still too few answers. Lancet Neurol. 12, 999–1010. doi: 10.1016/S1474-4422(13)70168-6, PMID: 23972913

[ref63] Török M. E. (2015). Tuberculous meningitis: advances in diagnosis and treatment. Br. Med. Bull. 113, 117–131. doi: 10.1093/bmb/ldv003 25693576

[ref64] Wang T. Feng G. D. Pang Y. Liu J. Y. Zhou Y. Yang Y. N.. (2016). High rate of drug resistance among tuberculous meningitis cases in Shaanxi province, China. Sci. Rep. 6:25251. doi: 10.1038/srep25251, PMID: 27143630 PMC4855176

[ref65] Wang T. Feng G. D. Pang Y. Yang Y. N. Dai W. Zhang L.. (2016). Sub-optimal specificity of modified Ziehl-Neelsen staining for quick identification of tuberculous meningitis. Front. Microbiol. 7:2096. doi: 10.3389/fmicb.2016.02096 28082963 PMC5186791

[ref66] Wang S. Yang W. Zhu M. Wang X. Pan L. Jin T.. (2023). Cerebrospinal fluid protein levels are elevated 100 times in a leptomeningeal metastasis patient: a case report and literature review. Front. Neurosci. 17:1174309. doi: 10.3389/fnins.2023.1174309, PMID: 37266544 PMC10229901

[ref67] Wilkinson R. J. Rohlwink U. Misra U. K. van Crevel R. Mai N. T. H. Dooley K. E.. (2017). Tuberculous meningitis. Nat. Rev. Neurol. 13, 581–598. doi: 10.1038/nrneurol.2017.120 28884751

[ref68] Woods G. L. Brown-Elliott B. A. Conville P. S. Desmond E. P. Hall G. S. Lin G.. Susceptibility Testing of Mycobacteria, Nocardiae, and Other Aerobic Actinomycetes [Internet]. 2nd ed. Wayne (PA): Clinical and Laboratory Standards Institute. (2011). 31339680

[ref69] World Health Organization (2010). Treatment of tuberculosis: guidelines. Geneva: World Health Organization. 23741786

[ref70] World Health Organization (2011). Guidelines for the programmatic Management of Drug-Resistant Tuberculosis: 2011 update. Geneva: World Health Organization. 23844450

[ref71] World Health Organization (2014, 2014). Xpert MTB/RIF Implementation Manual: Technical and operational ‘how-to’; practical considerations. Geneva: World Health Organization. 25473699

