# Supplementary material for: Disease spectrum and prognostic factors in patients treated for tuberculous meningitis in Shaanxi province, China
Source: Front Microbiol. 2024 May 17;15:1374458. doi: 10.3389/fmicb.2024.1374458 (PMC11140062; doi:10.3389/fmicb.2024.1374458)
Supplement: Supplementary file 1 [file Data_Sheet_1.docx]

**Panel S1. Diagnostic criteria and scores assigned by the uniform TBM research case definition^a^.**

| **Diagnostic criteria** | **Score** |
| --- | --- |
| **Clinical** (Maximum category score=6) | |
| Symptom duration of more than 5 days | 4 |
| Systemic symptoms suggestive of TB (1 or more of): weight loss/ (poor weight gain in children), night sweats or persistent cough >2 weeks | 2 |
| History of recent close contact with an individual with pulmonary TB or a positive TST/IGRA in a child <10 years | 2 |
| Focal neurological deficit (excluding cranial nerve palsies) | 1 |
| Cranial nerve palsy | 1 |
| **CSF** (Maximum category score=4) | |
| Clear appearance | 1 |
| Cells: 10–500 per μl | 1 |
| Lymphocytic predominance (>50%) | 1 |
| Protein concentration greater than 1 g/L | 1 |
| CSF to plasma glucose ratio of less than 50% or an absolute CSF glucose concentration less than 2.2mmol/L | 1 |
| **Cerebral imaging (CT and/or MRI)** (Maximum category score=6) | |
| Hydrocephalus | 1 |
| Basal meningeal enhancement | 2 |
| Tuberculoma | 2 |
| Infarct | 1 |
| Pre-contrast basal hyperdensity | 2 |
| **Extraneural TB (Maximum category score=4)** | |
| Chest radiograph suggestive of active TB (excludes miliary TB) | 2 |
| Chest radiograph suggestive of miliary TB | 4 |
| CT/ MRI/ US evidence of TB outside the CNS | 2 |
| AFB identified or *M. tuberculosis* cultured from another source i.e., sputum, lymph node, gastric washing, urine, blood culture | 4 |
| **Exclusion of alternative diagnoses-** An alternative diagnosis must be confirmed microbiologically, serologically or histopathologically | |
| **Definite TBM** = AFB seen on CSF microscopy, positive CSF *M. tuberculosis* culture, or positive CSF *M. tuberculosis* commercial NAAT in the setting of symptoms/signs suggestive of meningitis; or AFB seen in the context of histological changes consistent with TB brain or spinal cord together with suggestive symptoms/signs and CSF changes, or visible meningitis (on autopsy). | |
| **Probable TBM** = total score of ≥12 if neuroimaging available (total score of ≥10 if unavailable) | |
| **Possible TBM** = total score of 6-11 if neuroimaging available (total score of 6-9 if unavailable) | |
| **Not TBM=** Alternative diagnosis established, without a definitive diagnosis of tuberculous  meningitis or other convincing signs of dual disease. | |

TBM - tuberculous meningitis, TB- tuberculosis, TST - tuberculin skin test, IGRA - interferon gamma-release assay, CSF - cerebrospinal fluid, CT - computed tomography, MRI- magnetic resonance imaging, CNS - Central Nervous System; US - ultrasound, AFB -acid-fast bacilli, NAAT - nucleic acid amplification test

^a^ As described by Marais S, et.al. (1)

**Panel S2. The Modified Barthel Index ^a^ for activities of daily living.**

| **Patient’s independence** | **Score** |
| --- | --- |
| **Bowel control** | |
| Incontinent or needs enemas | 0 |
| Occasional accident (1x/week) | 1 |
| Continent | 2 |
| **Bladder control** | |
| Incontinent or needs enemas | 0 |
| Occasional accident (1x/week) | 1 |
| Continent | 2 |
| **Grooming** | |
| Needs help with personal care | 0 |
| Independent (including face, hair, teeth, shaving) | 1 |
| **Toilet use** | |
| Dependent | 0 |
| Needs some help | 1 |
| Independent | 2 |
| **Feeding** | |
| Unable | 0 |
| Needs help, e.g. cutting | 1 |
| Independent | 2 |
| **Transfers (bed to chair and back)** | |
| Unable, no sitting balance | 0 |
| Major help (1 or 2 people), can sit | 1 |
| Independent | 3 |
| **Mobility** | |
| Immobile | 0 |
| Wheelchair independent (including corners) | 1 |
| Walks with the help of 1 person (physical or verbal help) | 2 |
| Independent (may use aid) | 3 |
| **Dressing** | |
| Dependent | 0 |
| Needs help –can do ~½ unaided | 1 |
| Independent (including buttons, zips, laces,etc.) | 2 |
| **Stairs** | |
| Unable | 0 |
| Needs help (verbal or physical) | 1 |
| Independent | 2 |
| **Bathing** | |
| Dependent | 0 |
| Independent (bath or shower) | 1 |
| **Score interpretation** | |
| Independent (supervision or assistance may be needed) | 20 |
| Needs minimal help | 15 |
| Needs moderate help | 10 |
| Needs extensive help | 5 |
| Totally dependent (cannot perform any activities of daily living) | 0 |

^a^ The modified Barthel Index assesses a person's functional independence and the score is often used to evaluate a patient's level of dependence or independence and to track their progress over time. (2) We conducted outcome disability assessment of all the patients utilizing the modified Barthel Index 9-24 months post treatment. Clinical outcomes were categorized as poor if the score was ≤12, indicating a diminished functional independence.

**Panel S3. PCR primer sequences used in *Mycobacterium* species identification.**

| **Locus** |  |  | **PCR primer pairs^a^** | **Product size** |  | **Tm (℃)** |
| --- | --- | --- | --- | --- | --- | --- |
| ***16-23467*** |  |  | F 5’AGCCAGTGGCCTAACCCTCGG | 450bp |  | 61 |
|  |  |  | R 5’CCGAGGCATATCGCAGCCTCC |  |  |  |
| ***rrs1690*** |  |  | F 5’GGGGCGTGGCCGTTTGTTTT | 1800bp |  | 61 |
|  |  |  | R 5’CACCCGGCTCTCGCCCACTA |  |  |  |
| ***16s555*** |  |  | F 5’GGCGTGCTTAACACATGCAA | 500bp |  | 60 |
|  |  |  | R 5’TCACGAACAACGCGACAAAC |  |  |  |

^a^ F-forward primer; R-reverse primer.

**Panel S4. PCR primer sequences used in drug resistant gene sequencing.**

| **Drug** | **Locus** | **PCR primer pairs^a^** | **Product size** | **Tm(℃）** |
| --- | --- | --- | --- | --- |
| **Isoniazid** | ***InhA*** | F 5'-AgAAggTCAACgCCAAC | 934bp | 60 |
|  |  | R 5'-TgTgTgCAgCTCgAgTAACC |  |  |
|  | ***KatG*** | F 5'-AATCGCGCCGGGCAAA | 1000bp | 60 |
|  |  | R 5'-GGTCCCTGCGGTCAGC |  |  |
|  |  | F 5’-TGAGACAGTCAATCCCGATGC | 1015bp | 60 |
|  |  | R 5’-TAACAGCTGGCCCGACAAC |  |  |
|  |  | F 5’-GTTGCCGGCGAAAACAATCA | 701bp | 60 |
|  |  | R 5’-CGGGGTTATCGCCGATGT |  |  |
| **Rifampin** | ***rpoB*** | F 5'-GCCGGCCGAAACCGA | 1000bp | 60 |
|  |  | R 5’-CGTAGCGCTTCTCCTTGAA |  |  |
|  |  | F5’-GCTGTTGGACATCTACCGCAA | 1000bp |  |
|  |  | R5’-GAGGGCACGGTTGGCG |  | 60 |
|  |  | F5’-GTCTGAGGTGGACTACATGGAC | 1001bp | 60 |
|  |  | R5’- GGAACGGCATGTCCTCAA |  |  |
|  |  | F5’-GAAACGCAAGATCTCCGACG | 971bp | 60 |
|  |  | R5’ -TTGACGTCGAGCACGTAACT |  |  |
| **Ethambutol** | ***embB*** | F 5'-TGATATTCGGCTTCCTGCTC | 380bp | 58 |
|  |  | R 5’-ACCGCTCGATCAGCACATAG |  |  |
| **Quinolones** | ***gyrA*** | F 5'-GATGACAGACACGACGTTGC | 380bp | 58 |
|  |  | R 5'-GGGCTTCGGTGTACCTCAT |  |  |
|  | ***gyrB*** | F 5’-CCACCGACATCGGTGGATT | 420bp | 60 |
|  |  | R 5'-CTGCCACTTGAGTTTGTACA |  |  |
| **Injectables** | ***rrs-KAN^b^*** | F 5’- TATTAGACTGGCAGGGTCGC | 1536bp | 60 |
|  |  | R 5'- AAGTCCGAGTGTTGCCTCAG |  |  |
|  | ***eis*** | F 5'- TAGCACGGCCTTCAGAACTC | 1284bp | 60 |
|  |  | R 5'- GCCAGACACTGTCGTCGTAA |  |  |
|  | ***rpsL*** | F 5' CATGGCCGACAAACAGAACG | 479bp | 60 |
|  |  | R 5' CCGTAGACCGGGTCGTTG |  |  |
|  | ***gidB*** | F 5'CGATAGTTGAAGCCTGGCCC | 811bp | 61 |
|  |  | R 5'CGTCTCGAGAGCGGAGAATG |  |  |

^a^ F-forward primer; R-reverse primer. ^b^ *rrs-KAN* represented the amplified region containing the other hot spot conferring Kanamycin resistant in *rrs* gene.

**Table S1. Baseline characteristics of patients with confirmed TBM ^a^, comparing those with TBM score ≥12 and 6-11**

| **Characteristic** | **All**  **N=63 (%)** | **Confirmed TBM** | | **p-value** |
| --- | --- | --- | --- | --- |
|  |  | **TBM score** **≥12**  **N=46 (%)** | **TBM score 6-11**  **N=17 (%)** |  |
| Median age-years (IQR) | 26 (3-82) | 27 (3-82) | 24（3-64） | 0.333 |
| Female sex | 35 (55.6) | 25 (54.3) | 10 (58.8) | 0.750 |
| Median TBM score (IQR) ^b^ | 14 (6-20) | 15 (12-20) | 10 (6-11) | **<0.001** |
| Reported diabetes | 1 (1.6) | 0 | 1 (5.9) | 0.097 |
| **History** |  | | |  |
| Fever | 43 (68.3) | 31 (67.4) | 12 (70.6) | 0.809 |
| Headache | 48 (76.2) | 40 (87.0) | 8 (47.1) | **0.001** |
| Vomiting | 27 (42.9) | 24 (52.2) | 3 (17.6) | **0.014** |
| Neck stiffness | 24 (38.1) | 23 (50.0) | 1 (5.9) | **0.001** |
| Seizures | 6 (9.5) | 6 (13.0) | 0 | 0.117 |
| **BMRC grading ^c^** |  |  |  |  |
| I | 32 (50.8) | 32 (69.6) | 15 (88.2) | 0.131 |
| II | 21 (33.3) | 21 (45.7) | 2 (11.8) | **0.013** |
| III | 10 (15.9) | 10 (21.7) | 0 | **0.036** |
| **Imaging** |  | | |  |
| CXR suggestive of active cavitating disease | 27 (42.9) | 24 (52.2) | 3 (17.6) | **0.014** |
| CXR indicative of disseminated/miliary TB | 6 (9.5) | 6 (13.0) | 0 | 0.117 |
| Hydrocephalus | 20 (31.8) | 20 (43.5) | 0 | **0.001** |
| Brain infarcts | 15 (23.8) | 13 (28.3) | 2 (11.8) | 0.172 |
| Basal meningeal enhancement | 18 (28.6) | 18 (39.1) | 0 | **0.002** |
| Granulomas/tuberculomas | 1 (1.6) | 1 (2.2) | 0 | 0.540 |
| **CSF findings** |  | | |  |
| Median leukocyte count- cells/𝜇l (IQR) | 141.5 (0-6100) | 212 (2-6100) | 90 (0-683) | **0.015** |
| Leukocyte (50-500) (cells/𝜇l) | 43 (68.3) | 33 (71.7) | 10 (58.8) | **0.328** |
| Median lymphocytes-% (IQR) | 60.8 (1.0-98.0) | 59.0 (1.0-93.5) | 79.0 (17-98) | 0.142 |
| Median neutrophils-% (IQR) | 26.0 (0-94.5) | 32.3 (0-94.5) | 4.0 (0-70) | **0.011** |
| Neutrophils ≥ 25 (%) | 33 (52.4) | 28 (60.9) | 5 (29.4) | **0.026** |
| Median monocytes-% (IQR) | 8.5 (0-53) | 7.5 (0-27) | 13.0 (2-53) | **0.013** |
| Median protein-mg/dl (IQR) | 1.5 (0.2-7.4) | 1.5 (0.2-7.4) | 0.6 (0.3-1.8) | **0.012** |
| Protein >1.0 mg/dl | 37 (58.7) | 34 (73.9) | 3 (17.6) | **<0.001** |
| Median glucose-mmol/l (IQR) | 1.8 (0.4-4.5) | 1.8 (0.5-4.5) | 1.9 (0.4-3.8) | 0.905 |
| Glucose < 2.2 mmol/l | 37 (58.7) | 29 (63.0) | 8 (47.1) | 0.253 |
| Median intracranial pressure (mmH_2_O) (IQR) | 260 (100-400) | 280 (100-400) | 260 (130-400) | 0.501 |
| **Any-drug resistance (%)** | 16 (25.4) | 13 (28.3) | 3 (17.6) | 0.390 |
| **Beijing genotype (%)** | N=25 (39.7) | N=16 (34.8) | N=9 (52.9) | 0.191 |
|  | 20 (80.0) | 12 (75.0) | 8 (88.9) | 0.405 |
| **Outcome** | N=60 (95.2) | N=46 (100.0) | N=14 (82.4) | **0.004** |
| TBM treatment completed ^d^ | 50 (83.3) | 39 (84.8) | 11 (78.6) | 0.585 |
| Lost to follow up | 3 (4.8) | 0 | 3 (21.4) | **0.001** |
| Death (on treatment) | 5 (8.3) | 4 (8.7) | 1 (7.1) | 0.854 |
| Death (post treatment) ^e^ | 12 (20.0) | 12 (26.1) | 0 | **0.033** |
| Alive with poor outcome ^f^ | 6 (10.0) | 6 (13.0) | 0 | 0.154 |
| Median Barthel score (IQR) ^g^ | 14 (2-20) | 14 (2-20) | 14 (14-20) | **0.008** |
| All poor outcome ^h^ | 23 (38.3) | 22 (47.8) | 1 (7.1) | **0.006** |

CXR-chest X-ray; TBM-tuberculous meningitis; TB-tuberculosis; CSF-cerebral spinal fluid; BMRC- British Medical Research Council; IQR-interquartile range.

Continuous variables are presented as median (interquartile range), and categorical variables as counts (proportions). Statistical differences between confirmed TBM cases (as defined) with consensus TBM score of ≥12 and 6-11 points were assessed by χ^2^-test (categorical variables) and Mann-Whitney U test (continuous variables).

^a^ Confirmed TBM defined by a positive *M. tuberculosis* culture or Xpert MTB/RIF on CSF (1).

^b^ According to consensus uniform research case definition criteria (1) (see **Panel S1**).

^d^ Patients were treated for TBM for a period of 9-12 months.

^c^ TBM severity grade according to the revised British Medical Research Council disease severity grade (BMRC 1948) with stage 3 being most severe. (3)

^e^ Assessed 9-24 months post treatment completion

^f^ Modified Barthel Index score <12 (excluding dead); assessed 9-24 months post treatment completion

^g^ See **Panel S2** for calculation of Modified Barthel Index score (excluding dead). (2)

^h^ Death during or after treatment or Barthel index score <12; assessed 9-24 months after treatment completion. Only reported for those in whom an outcome was reported; lost to follow up excluded.

**Table S2. ROC curve on AUC values of TBM score ≥12 and MZN positive with confirmed TBM as the reference standard in 341 presumptive TBM patients who had at least a CSF culture or Xpert MTB/RIF performed.**

| **Predicted variable** | **Risk factors** | **AUC** | **Cut-off value** | **P** | **95%CI** | **Sensitivity (%)** | **Specificity (%)** |
| --- | --- | --- | --- | --- | --- | --- | --- |
| **Culture and/or Xpert positive patients** | TBM score ≥12 | 0.74 | 0.49 | <0.001 | 0.67-0.81 | 73.0 | 75.5 |
|  | MZN positive | 0.71 | 0.43 | <0.001 | 0.66-0.78 | 95.2 | 48.2 |
|  | TBM score of ≥ 12 & MZN | 0.81 | 0.49 | <0.001 | 0.76-0.86 | 70.0 | 79.1 |

AUC - area under the curve; CI - confidence interval; ROC-receiver operating characteristic; MZN - modified Ziehl-Neelsen staining.

**Table S3. Multivariable logistic regression analysis of risk factors for poor outcome ^a^ in 60 patients with confirmed TBM ^b^ who completed follow-up**

| **Characteristic** | **Outcome poor** | **Outcome not poor**  **N=37 (%)** | **Univariate** | | **Multivariate model** | |
| --- | --- | --- | --- | --- | --- | --- |
|  | **N=23 (%)** |  | **OR (95%CI)** | **P** | **Adjusted OR (95% CI)** | **P** |
| **Median TBM score (IQR) ^c^** | 15 (10-20) | 13 (6-18) | **1.454 (1.141-1.852)** | **0.002** | - | - |
| ≥15 | 14 (60.9) | 10 (27.0) | **4.200 (1.387-12.719)** | **0.011** | **-** | 0.336 |
| ≥12 | 22 (95.7) | 24 (64.9) | **11.917 (1.438-98.747)** | **0.022** | **-** | 0.545 |
| **BMRC grading ^d^** | - | | | | | |
| I | 7 (30.4) | 22 (59.5) | **0.298 (0.099-0.900)** | **0.032** | **-** | - |
| III | 7 (30.4) | 3 (8.1) | **4.958 (1.132-21.722)** | **0.034** | - | 0.162 |
| **Imaging** | - | | | | | |
| Hydrocephalus | 13 (56.5) | 7 (18.9) | **5.571（1.738-17.856）** | **0.004** | **-** | 0.073 |
| Basal meningeal enhancement | 11 (47.8) | 7 (18.9) | **3.929 (1.231-12.535)** | **0.021** | - | 0.903 |
| CXR suggestive of active cavitating disease | 10 (43.5) | 17 (45.9) | **-** | 0.852 | - | - |
| CXR indicative of disseminated/miliary TB | 5 (21.7) | 1 (2.7) | **10.000 (1.086-92.104)** | **0.042** | **19.183 (1.601-229.896)** | **0.020** |
| **CSF findings** | - | | | | | |
| Median neutrophils-% (IQR) | 38.0 (0.0-78.0) | 12.5 (0-94.5) | **-** | 0.101 | - | - |
| Intracranial pressure (mmH_2_O) | 300 (115-400) | 260 (100-400) | **-** | 0.282 | - | - |
| Any-drug resistance | 5 (21.7) | 11 (29.7) | **-** | 0.498 | **-** | **-** |

CXR-chest X-ray; OR - odds ratio; CI - confidence interval; BMRC grade - British Medical Research Council disease severity grade; TBM - tuberculous meningitis; CSF - cerebral spinal fluid; TB - tuberculosis; IQR-interquartile range. Age, female, fever, headache, vomiting, neck stiffness, seizures, BMRC grading II, chest X-ray suggestive of active cavitating disease, infarcts, CSF leukocyte, CSF lymphocyte, CSF monocytes, CSF neutrophils, CSF protein, CSF glucose, intracranial pressure and any-drug resistance were non-significant controlled covariates.

Continuous variables are presented as median (IQR), and categorical variables are presented as counts (proportions).

^a^ Death during or after treatment or Barthel index score <12; assessed 9-24 months after treatment completion (see **Panel S2**) (2). Three patients with confirmed TBM who were lost to follow-up were excluded from the comparative analysis.

^b^ Confirmed TBM defined by a positive *M. tuberculosis* culture or Xpert MTB/RIF on CSF (1) (**Table S1**).

^c^ According to consensus uniform research case definition criteria (1) (**Table S1**).

^d^ TBM severity grade according to the revised British Medical Research Council disease severity grade (BMRC 1948) with stage 3 being most severe. (3)

**Table S4. Multivariable logistic regression analysis of risk factors for death ^a^ in 60 patients with confirmed tuberculous meningitis ^b^ who completed follow-up**

| **Characteristic** | **Death** | **Alive** | **Univariate** | | **Multivariate model** | |
| --- | --- | --- | --- | --- | --- | --- |
|  | **N=17 (%)** | **N=43 (%)** | **OR (95%CI)** | **P** | **Adjusted OR (95% CI)** | **P** |
| **Median age-years (IQR)** | 41 (3-82) | 25 (3-64) | **1.032 (1.001-1.064)** | **0.046** | **-** | **-** |
| <15 | 3 (17.6) | 4 (9.3) | - | 0.372 | **-** | **-** |
| 15-60 | 9 (52.9) | 22 (51.2) | - | 0.901 | **-** | **-** |
| >60 | 4 (23.5) | 2 (4.7) | **6.308 (1.034-38.481)** | **0.046** | - | 0.110 |
| **Median TBM score (IQR) ^c^** | 16 (10-20) | 13 (6-18) | **1.466 (1.127-1.906)** | **0.004** | - | - |
| ≥15 | 12 (70.6) | 12 (27.9) | **6.200 (1.798-21.380)** | **0.004** | **8.437 (1.328-53.585)** | **0.024** |
| ≥12 | 16 (94.1) | 30 (69.8) | **-** | 0.074 | - | - |
| **BMRC grading ^d^** | - | | | | | |
| I | 6 (35.3) | 23 (53.5) | - | 0.208 | - | - |
| II | 5 (29.4) | 16 (37.2) | - | 0.569 | - | - |
| III | 6 (35.3) | 4 (9.3) | **5.318 (1.271-22.250)** | **0.022** | - | 0.460 |
| **Imaging** |  | | | | | |
| Hydrocephalus | 9 (52.9) | 11 (25.6) | **3.273（1.013-10.578）** | **0.048** | - | 0.691 |
| Basal meningeal enhancement | 9 (52.9) | 9 (20.9) | **4.250 (1.276-14.151)** | **0.018** | - | 0.828 |
| CXR suggestive of active cavitating disease | 6 (35.3) | 21 (48.8) | **-** | 0.345 | - | - |
| CXR indicative of disseminated/miliary TB | 4 (23.5) | 2 (4.7) | **6.308 (1.034-38.481)** | **0.046** | **12.427 (1.138-135.758)** | **0.039** |
| **CSF findings** |  | | | | | |
| Median lymphocytes-% (IQR) | 46.8 (2-86.5) | 70.5 (1.0-98.0) | 0.978 (0.958-0.999) | 0.045 | - | - |
| Lymphocytes >75 (%) | 3 (17.6) | 21 (48.8) | 0.242 (0.060-0.971) | **0.045** | - | - |
| Median neutrophils-% (IQR) | 40.0 (0-78.0) | 12.0 (0-94.5) | 1.020 (1.000-1.041) | 0.050 | - | - |
| Neutrophils ≥ 25 (%) | 14 (82.4) | 18 (41.9) | **6.481 (1.620-25.927)** | **0.008** | - | 0.296 |
| Intracranial pressure (mmH_2_O) | 300 (115-400) | 258 (100-400) | **-** | 0.143 | - | - |
| Any-drug resistance | 4 (23.5) | 12 (27.9) | **-** | 0.730 | **-** | **-** |

CXR-chest X-ray; OR - odds ratio; CI - confidence interval; BMRC grade - British Medical Research Council disease severity grade; TBM - tuberculous meningitis; CSF - cerebral spinal fluid; TB – tuberculosis; IQR-interquartile range. Female, fever, headache, vomiting, neck stiffness, seizures, BMRC grading (I, II), chest X-ray suggestive of active cavitating disease, infarcts, granulomas/tuberculomas, CSF leukocyte, CSF monocytes, CSF protein, CSF glucose, intracranial pressure and any-drug resistance were non-significant controlled covariates.

Continuous variables are presented as median (IQR), and categorical variables are presented as counts (proportions).

^a^ Death during or after treatment or Barthel index score <12; assessed 9-24 months after treatment completion (see **Panel S2**) (2). Three patients with confirmed TBM who were lost to follow-up were excluded from the comparative analysis.

^b^ Confirmed TBM defined by a positive *M. tuberculosis* culture or Xpert MTB/RIF on CSF (1) (**Table S1**).

^c^ According to consensus uniform research case definition criteria (1) (**Table S1**).

^d^ TBM severity grade according to the revised British Medical Research Council disease severity grade (BMRC 1948) with stage 3 being most severe. (3)

**Table S5. Multivariable logistic regression analysis for risk factors of poor outcome^a^ in 210** **probable & possible TBM patients who completed follow-up**

| **Characteristic** | **Univariate** | **Multivariate model** | | |
| --- | --- | --- | --- | --- |
|  | **OR (95%CI)** | **P** | **Adjusted OR (95% CI)** | **P** |
| Headache | **0.357 (0.168-0.757)** | **0.007** | **-** | 0.147 |
| Seizures | **2.829 (1.283-6.237)** | **0.010** | **-** | 0.315 |
| BMRC grading ^b^ |  | | | |
| I | **0.122 (0.042-0.358)** | **<0.001** | **0.240 (0.076-0.762)** | **0.015** |
| III | **7.947 (3.635-17.374)** | **<0.001** | **5.129 (2.152-12.222)** | **<0.001** |
| CXR suggestive of active cavitating disease | **-** | 0.536 | - | **-** |
| CXR indicative of disseminated/miliary TB | - | 0.542 | - | **-** |
| Intracranial pressure (mmH2O) | - | 0.063 | - | **-** |

CXR-chest X-ray; TBM-tuberculous meningitis; TB - tuberculosis; OR - odds ratio; CI - confidence interval; BMRC grade - British Medical Research Council disease severity grade; Age, female, fever, vomiting, neck stiffness, BMRC grade II, diagnostic score, CXR suggestive of active cavitating disease or disseminated/miliary TB, hydrocephalus, infarcts, basal meningeal enhancement, granulomas/tuberculomas, CSF leukocyte, CSF neutrophils, CSF monocytes, CSF protein, CSF glucose and intracranial pressure were non-significant controlled covariates.

^a^ Death during or after treatment or Barthel index score <12; assessed 9-24 months after treatment completion (see **Panel S2**). (2) 19 patients with probable & possible TBM patients who were lost to follow-up were excluded from the comparative analysis.

^b^ TBM severity grade according to the revised British Medical Research Council disease severity grade (BMRC 1948) with stage 3 being most severe. (3)

**Table S6. Drug-resistance, genotype and outcome in culture-confirmed TBM patients who had phenotypic drug susceptibility testing and sequencing done (N=25)**

| **Individual drug** | **Phenotypic resistance** (Strain No.) | **Resistance-associated mutations** | | | **MIC^a^**  **(Critical concentration^b^)** μg/ml | **Resistance detected by Xpert MTB/RIF** | **Outcome** | **Genotypes** |
| --- | --- | --- | --- | --- | --- | --- | --- | --- |
|  |  | **Genes** | **Nucleotide change ^g^** | **Amino acid change** |  |  |  |  |
| **Isoniazid** (4) | H, R, S (#11) | ***kat G*** | 315, AGC→ACC | S→T | >32.0 (4.0) | **R** | Good | Beijing |
|  |  |  | 463, CGG→CTG | R→L^c^ |  |  |  |  |
|  | H, R, E (#18) |  | 315, AGC→ACC | S→T | >32.0 (4.0) | - | Good | Beijing |
|  |  |  | 463, CGG→CTG | R→L |  |  |  |  |
|  | H, R, C, A, K (#19) |  | 315, AGC→ACC | S→T | >32.0 (4.0) | - | Good | Beijing |
|  |  |  | 463, CGG→CTG | R→L |  |  |  |  |
|  | H (#27) |  | 315, AGC→ACC | S→T | >32.0 (4.0) | - | Good | Beijing |
|  |  |  | 463, CGG→CTG | R→L |  |  |  |  |
|  | - | ***inhA* or promotor** | - | - | - | - | - |  |
| **Rifampin** (4) | H, R, S (#11) | ***rpoB*** | 430, CTG→CCG^e^ | L→P | 2.0 (1.0) | - | Good | Beijing |
|  | H, R, E (#18) |  | 450, TCG→TTG^e^ | S→L | >8.0 (1.0) | - | Good | Beijing |
|  | H, R, C, A, K (#19) |  | 452, CTG→CCG^e^ | L→P | 8.0 (1.0) | - | Good | Beijing |
|  | R (#15) ^d^ |  | - | - | 2.0 (1.0) | **R** | Good | T2 |
| **Ethambutol** (2) | H, R, E (#18) | ***embB*** | 306, ATG→ATA | M→I | 20.0 (5.0) | - | Good |  |
|  | E (#2) ^d^ |  | - | - | >20.0 (5.0) | - | Good |  |
| **Quinolones** (0) | H, R, E (#18) | ***gyrA*** | 95, AGC→ACC^f^ | S→T | Mfx-0.3 (2.0)  Lfx -0.6 (2.0) | - | Good | Beijing |
|  |  |  | 90, GCG→GTG | A→V |  |  |  |  |
|  | - | ***gyrB*** | - | - | - | - | - |  |
| **Injectables** (6) | H, R, **S** (#11) | ***rpsL*** | 43, AAG→AGG | K→R | S>4.0 (2.0)  K-4.0 (5.0)  A-2.0 (4.0)  C-2.5 (5.0) | - | Good | Beijing |
|  |  | ***gidB*** | 92, GAA→GAC^c^  205, GCA→GCG^c^ | E→D  A→A |  |  |  |  |
|  | **S** (#14) | ***rpsL*** | 43, AAG→AGG | K→R | S>4.0 (2.0)  K-4.0 (5.0)  A-2.0 (4.0)  C-5.0 (5.0) | - | Good | Beijing |
|  |  | ***gidB*** | 92, GAA→GAC  205, GCA→GCG | E→D  A→A |  |  |  |  |
|  | **S** (#1) | ***gidB*** | 92, GAA→GAC  205, GCA→GCG | E→D  A→A | S>4.0 (2.0)  K-8.0 (5.0)  A-4.0 (4.0)  C-5.0 (5.0) | - | **Dead** | Beijing |
|  | K (#9) | ***gidB*** | 16, CTT→CGT | L→R | S-2.0 (2.0)  K-16.0 (5.0)  A-8.0 (4.0)  C-5.0 (5.0) | - | **Dead** | LAM6 |
|  | H, R, S, C, A, K (#19) | ***gidB*** | 92, GAA→GAC  205, GCA→GCG | E→D  A→A | S>4.0 (2.0)  K>128.0 (5.0) A >32.0 (4.0)  C-20.0 (5.0) | **R** | Good | Beijing |
|  |  | ***rrs-KAN*** | 1401, A→G | - |  |  |  |  |
|  | C, A, K (#26) | ***-*** | - | - | S-4.0 (2.0)  K-32.0(5.0)  A-8.0 (4.0)  C-20.0 (5.0) | - | Good | T1 |
|  | -(#16) | ***rpsL*** | 39, ACC→ACT | T→T | S-2.0 (2.0)  K-8.0(5.0)  A-1.0 (4.0)  C-5.0 (5.0) | - | **Dead** | Beijing |
|  |  | ***gidB*** | 92, GAA→GAC  205, GCA→GCG | E→D  A→A |  |  |  |  |
|  | H (#27) | ***rpsL*** | 43, AAG→AGG | K→R | S-2.0 (2.0)  K-4.0(5.0)  A-2.0 (4.0)  C-5.0 (5.0) | **R** | Good | Beijing |
|  |  | ***gidB*** | 92, GAA→GAC  205, GCA→GCG | E→D  A→A |  |  |  |  |
|  | **-** | ***eis*** | - | - | - | - | - |  |

H- isoniazid; R- rifampin; E- ethambutol; Mfx-moxifloxacin; Lfx-levofloxacin; S-streptomycin; K- kanamycin, A-amikacin; C-capreomycin; RRDR-Rifampin Resistance Determining Region; DST- drug sensitivity testing; WHO- World Health Organization; CLSI- Clinical and Laboratory Standards Institute.

^a^MIC-Mean Inhibitory Concentration; ^b^ Critical concentration as recommended by WHO and CLSI (4); ^c^ Detected in all 20 Beijing and 1 atypical Beijing strain; ^d^ Resistance identified by L-J solid DST and MGIT 960 MIC; ^e^ L511P, S531L, L533P with *Escherichia coli* numbering (all within the 81 base pair RRDR); ^f^ Detected in all the isolates, both drug resistant and drug susceptible; ^g^ compared to H37Rv. Sequences were compared to the *M. tuberculosis* reference strain H37Rv and deposited in GenBank (<http://www.ncbi.nlm.nih.gov/BLAST/>) (**Panel S4**).

**References**

1. Marais S, Thwaites G, Schoeman JF, Török ME, Misra UK, Prasad K, et al. Tuberculous meningitis: a uniform case definition for use in clinical research. Lancet Infect Dis. 2010;10(11):803-12.

2. Collin C, Wade DT, Davies S, Horne V. The Barthel ADL Index: a reliability study. Int Disabil Stud. 1988;10(2):61-3.

3. Solomons RS, Visser DH, Donald PR, Marais BJ, Schoeman JF, van Furth AM. The diagnostic value of cerebrospinal fluid chemistry results in childhood tuberculous meningitis. Childs Nerv Syst. 2015;31(8):1335-40.

4. Woods GL, Brown-Elliott BA, Conville PS, Desmond EP, Hall GS, Lin G, et al. Susceptibility Testing of Mycobacteria, Nocardiae, and Other Aerobic Actinomycetes. Wayne PA: ©2011 Clinical and Laboratory Standards Institute.; Except as stated below, any reproduction of content from a CLSI copyrighted standard, guideline, companion product, or other material requires express written consent from CLSI Interested parties may send permission requests to permissions@clsi.org.; CLSI hereby grants permission to each individual member or purchaser to make a single reproduction of this publication for use in its laboratory procedure manual at a single site. To request permission to use this publication in any other manner, e-mail permissions@clsi.org.; 2011 Mar.
