# Supplementary figures and images for: Disease spectrum and prognostic factors in patients treated for tuberculous meningitis in Shaanxi province, China
Source: Front Microbiol. 2024 May 17;15:1374458. doi: 10.3389/fmicb.2024.1374458 (PMC11140062; doi:10.3389/fmicb.2024.1374458)

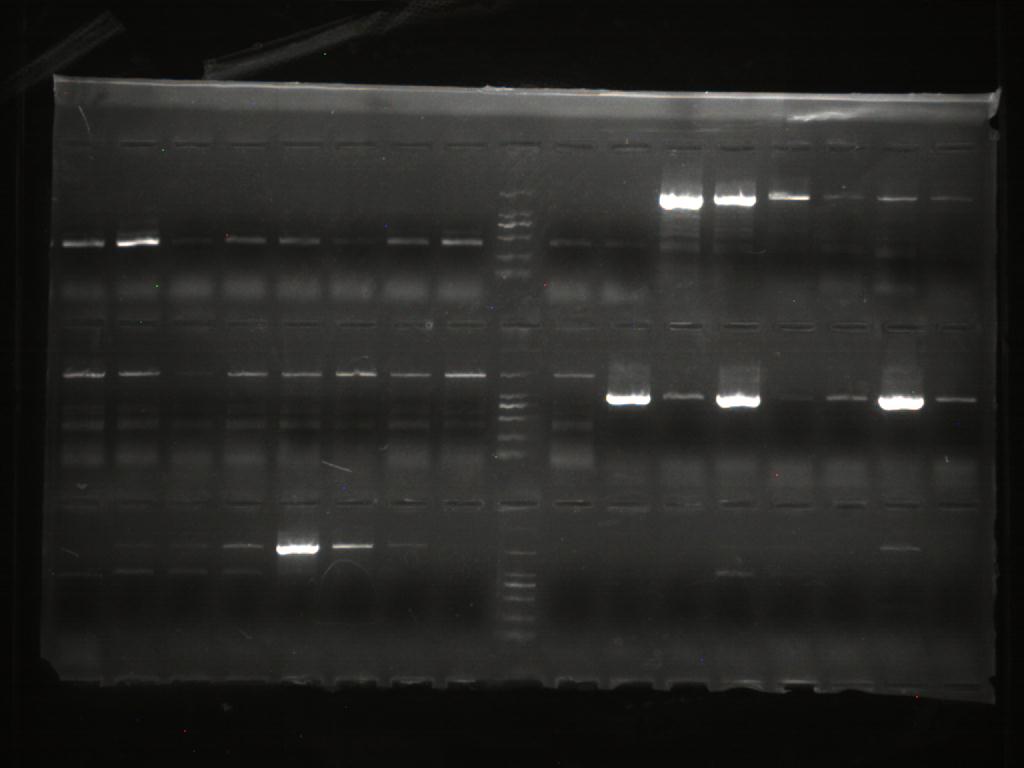

Supplement: Supplementary file 11 [file Data_Sheet_11.zip › ΦÇÉΦì»σƒ║σ¢á6/20130509-T0009/τÄïσ⌐╖ 0531Φâ╢σ¢╛σÅèμÿÄτ╗å/τÄïσ⌐╖ 0531Φâ╢σ¢╛σÅèμÿÄτ╗å/20130531-σêçΦâ╢2-1.jpg]

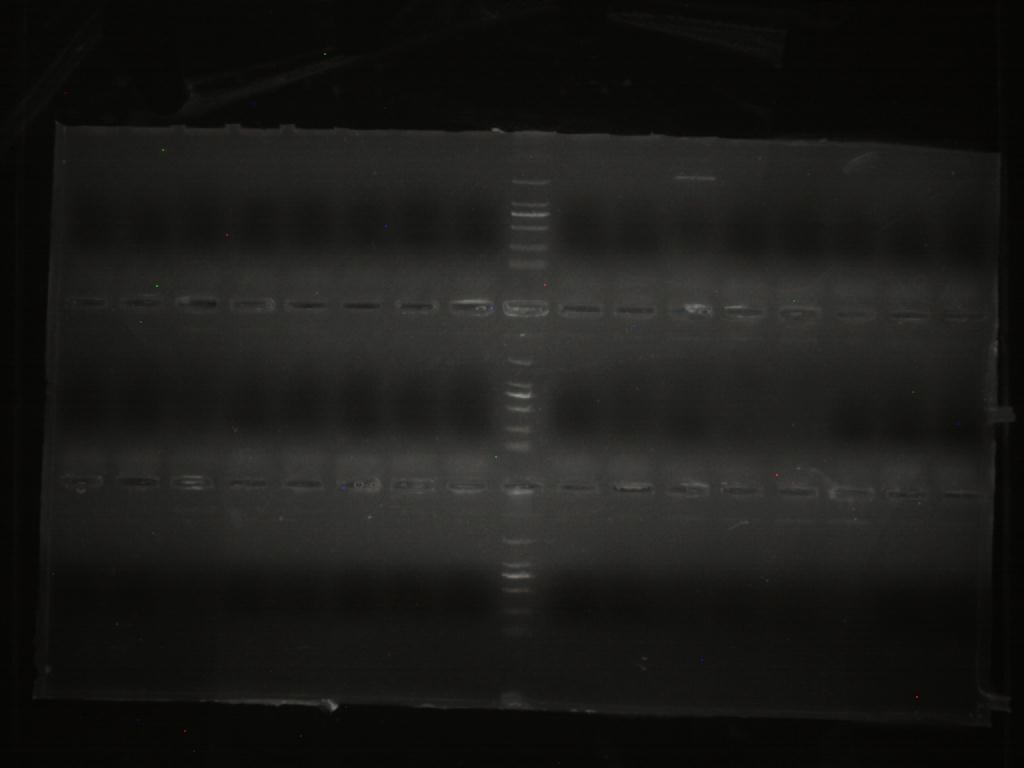

Supplement: Supplementary file 11 [file Data_Sheet_11.zip › ΦÇÉΦì»σƒ║σ¢á6/20130509-T0009/τÄïσ⌐╖ 0531Φâ╢σ¢╛σÅèμÿÄτ╗å/τÄïσ⌐╖ 0531Φâ╢σ¢╛σÅèμÿÄτ╗å/20130531-σêçΦâ╢3-1.jpg]

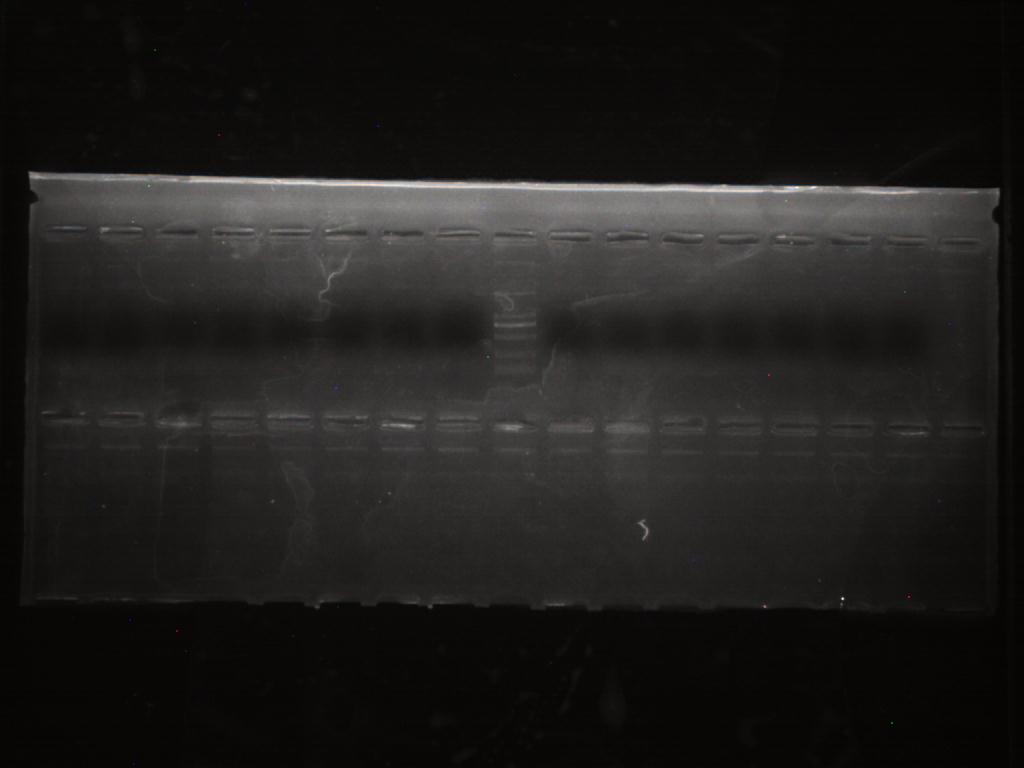

Supplement: Supplementary file 11 [file Data_Sheet_11.zip › ΦÇÉΦì»σƒ║σ¢á6/20130509-T0009/τÄïσ⌐╖ 0531Φâ╢σ¢╛σÅèμÿÄτ╗å/τÄïσ⌐╖ 0531Φâ╢σ¢╛σÅèμÿÄτ╗å/20130531-σêçΦâ╢4-1.jpg]
